# Bioprospecting Microbial Diversity for Lignin Valorization: Dry and Wet Screening Methods

**DOI:** 10.3389/fmicb.2020.01081

**Published:** 2020-06-09

**Authors:** Carolyne Caetano Gonçalves, Thiago Bruce, Caio de Oliveira Gorgulho Silva, Edivaldo Ximenes Ferreira Fillho, Eliane Ferreira Noronha, Magnus Carlquist, Nádia Skorupa Parachin

**Affiliations:** ^1^Department of Genomic Science and Biotechnology, Universidade Católica de Brasília – UCB, Brasília, Brazil; ^2^Laboratory of Enzymology, Department of Cellular Biology, University of Brasília, Brasília, Brazil; ^3^Division of Applied Microbiology, Department of Chemistry, Faculty of Engineering, Lund University, Lund, Sweden

**Keywords:** lignin-degrading enzymes, biosensors, activity-based screening, bioprocess, high-throughput screening

## Abstract

Lignin is an abundant cell wall component, and it has been used mainly for generating steam and electricity. Nevertheless, lignin valorization, i.e. the conversion of lignin into high value-added fuels, chemicals, or materials, is crucial for the full implementation of cost-effective lignocellulosic biorefineries. From this perspective, rapid screening methods are crucial for time- and resource-efficient development of novel microbial strains and enzymes with applications in the lignin biorefinery. The present review gives an overview of recent developments and applications of a vast arsenal of activity and sequence-based methodologies for uncovering novel microbial strains with ligninolytic potential, novel enzymes for lignin depolymerization and for unraveling the main metabolic routes during growth on lignin. Finally, perspectives on the use of each of the presented methods and their respective advantages and disadvantages are discussed.

## Introduction

Lignin is one of the most abundant macromolecules available as a raw material for biorefining. However, it is also one of the most underused plant constituents in biorefineries and is now primarily used for generating process steam and electricity (Y.-H. P. Zhang, 2008; Ragauskas et al., 2014). The predominant reason is the inherent recalcitrance and structural heterogeneity of lignin, making it challenging to access the valuable aromatic substituents. Nevertheless, *lignin valorization*, i.e. conversion into value-added fuels, chemicals, or materials, is considered crucial for the full implementation of cost-effective lignocellulosic biorefineries (Naseem et al., 2016).

**FIGURE 1:**
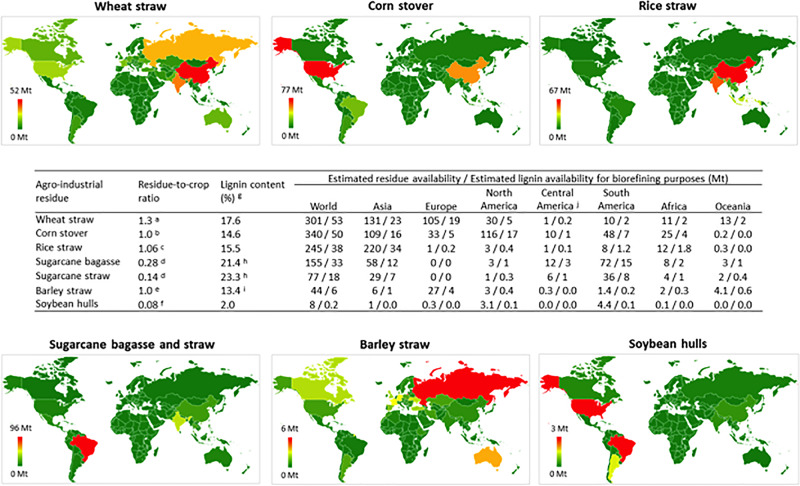
Global distribution and potential availability of major agro-industrial residues and lignin for valorization purposes. The global agricultural production in 2017 was obtained from the Food and Agriculture Organization of the United Nations (2019) Database (9), ^a^(5), ^b^(10), ^c^(11), ^d^(12), ^e^(13), ^f^(14), ^g^(15), ^h^(16), and ^I^(17). Figure footnotes: The global production of wheat, corn, rice, sugarcane, soybeans and barley in 2017 was obtained from the Food and Agriculture Organization of the United Nations (2019) Database (http://www.fao.org/faostat/en/#data) and was used as input data to calculate the availability of crop residues and lignin for biorefining purposes. The total amount of residue generated from the processing of each crop (wheat straw, corn stover, rice straw, sugarcane bagasse and straw, soybean hulls and barley straw) was calculated by multiplying the total crop amount by the “residue-to-crop ratio”. The “residue-to-crop ratio” for each crop was calculated elsewhere and is available in the literature, as indicated in the figure. The realistic availability of crop residues for biorefining purposes was estimated by multiplying the total amount of crop residues by 0.3 [i.e. corresponding to 30% of the total, as estimated by Daioglou et al. (2016)]. Last, the availability of lignin for biorefining purposes was calculated by multiplying the estimated realistic availability of crop residues for biorefining purposes by the lignin content of each residue (as obtained from the literature, as indicated in the figure). The raw data employed to build the figure are shown in Supplementary Material.

Lignin can be extracted using a range of different processes resulting in different types of TLs, with Kraft lignin, lignosulfonates, soda lignin, and organosolv lignin being the most prevalent (Ragauskas et al., 2014; Bajwa et al., 2019). Historically, the wood pulping industry has been the primary source of TLs; however, the growing cellulosic ethanol industry is using agro-industrial residues as a feedstock, which is also becoming a significant source of lignin^1^. Annually, agro-industrial residues are generated in enormous amounts globally, and they typically contain approximately 15–25% lignin (Kadam and McMillan, 2003; Strassberger et al., 2014). Residues from six major commodity crops, namely, wheat straw, rice straw, corn stove, barley straw, sugarcane bagasse and straw and soybean hulls, provided a total of approximately 3.9 Gt of biomass in 2017. The high availability of agro-industrial waste biomass worldwide (Figure 1) represents an opportunity to develop technologies for producing bio-based fuels, chemicals, and materials (Silva et al., 2018).

**FIGURE 2:**
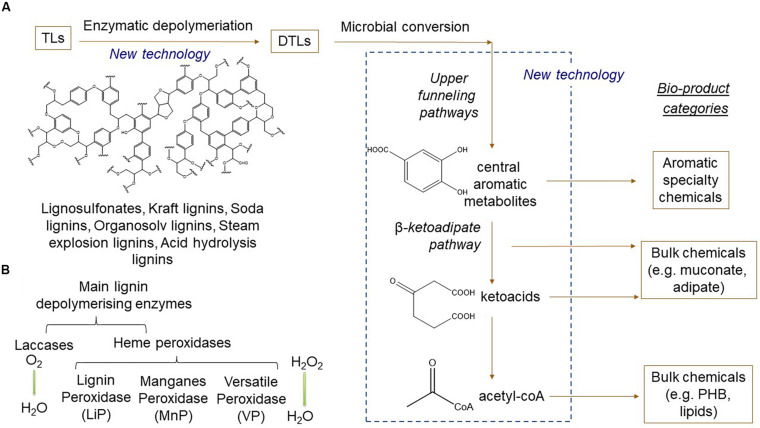
Schematic overview of novel technologies for bioprocessing technical lignins (TLs) into specialty and bulk chemicals. **(A)** Technical lignins are depolymerized by lignolytic enzymes into depolymerized technical lignins (DTLs) consisting in a mixture of monomeric and dimeric aromatic compounds. DTLs are further converted by microorganisms via upper funneling pathways into central aromatic branching nodes (e.g. catechol or protocatechuate acid). These compounds are converted by different metabolic pathways depending on the target bioproduct category, as exemplified here with aromatic specialty chemicals, ketoacid or acetyl-coA-derived precursors for polymers, e.g. bioplastics or lipids. **(B)** Primary lignin depolymerizing enzymes categorized by family and type of substrate.

A possible route to lignin valorization that has attracted considerable interest in recent years is combined chemo-catalytic or enzymatic conversion TLs into depolymerized technical lignins (DTLs) and subsequent bio-catalytic processing using microbial cell factories [see reviews by Abdelaziz et al. (2016), Beckham et al. (2016), Sun et al. (2018), Ponnusamy et al. (2019), and Becker and Wittman (2019)]; (Figure 2). For efficient enzymatic or microbial production of value-added chemicals, TLs need to be depolymerized to higher titers and ideally into a homogenous blend of compounds, which is challenging considering the inherent recalcitrance and heterogeneity of the polymer. A variety of chemical depolymerization approaches, such as hydrolysis, hydrogenolysis, gasification, and thermochemical oxidation, have previously been developed (Welker et al., 2015; Hämäläinen et al., 2018; Liu et al., 2018; Xu R. et al., 2018), resulting in a heterogenous mixture of aromatic compounds. Further information can be found in recent reviews concerning chemical TL depolymerization from a biorefinery perspective (Ragauskas et al., 2014) and from a chemo-catalytic perspective (Behling et al., 2016; Gillet et al., 2017). Enzymatic approaches concerning lignin depolymerization have also been studied (Abdelaziz et al., 2016; Xu et al., 2019), although significant improvements remain before it can be a competitive alternative to chemo-catalytic methods. In this context, the discovery and engineering of novel oxidative enzymes, such as laccases and peroxidases, play a crucial role (Abdelaziz et al., 2016; Hämäläinen et al., 2018).

The aim of this review is to describe and compare dry (sequence-based) and wet (activity- and growth-based) methodologies available for identifying specific lignin-degrading activities. First, a short overview of the microbial utilization of lignin is given. Then, recent developments and applications of a vast arsenal of activity and sequence-based methodologies for discovering novel microbial strains with ligninolytic potential, novel enzymes for lignin depolymerization and for unraveling the primary metabolic routes during growth on lignin are reviewed. From the available literature on biological lignin valorization, it becomes clear that the selection of screening methods to draw specific activities toward the TLs and DTLs has to be well planned to succeed in the construction of novel industrial bioprocesses. This holds true regardless of whether the sources to be screened are natural microbial isolates, enrichment cultures or the diversity created by a synthetic biology approach (Abdelaziz et al., 2016; Helm et al., 2018; Xu et al., 2019). In this review, perspectives on using different screening methods and their respective advantages and disadvantages are discussed.

## Microbial Utilization of Lignin and Its Derivatives

White and brown rot fungi are widely known for being the primary microorganisms that act in lignin deconstruction (Lee, 1997). They are known to produce a variety of enzymes that depolymerize lignin, such as laccases (EC 1.10.3.2), lignin peroxidases (LiPs) (EC 1.11.1.14), MnPs (EC1.11.1.13) and versatile peroxidases (VPs) (EC 1.11.1.16) (Figure 2B). The white rot fungus *Pleurotus* sp. is known for producing different types of peroxidases to metabolize phenolic compounds such as veratryl alcohol, methoxybenzene, and benzoic acid (Gutiérrez et al., 1994; Guillén et al., 1997; Martínez, 2002; Martínez et al., 2005). The brown rot fungus *Coniophora puteana* has been described as a laccase producer, and it is able to degrade cell wall layers in response to the presence of tannic acid (K. H. Lee et al., 2004). The action of laccases and peroxidases results in the depolymerization of the lignin polymer in aromatic compounds, which can be converted to high-valued industrial products by bacteria (Kameshwar et al., 2018; Kameshwar and Qin, 2018; Bugg et al., 2012).

In contrast to fungi, the enzymes responsible for degrading the lignin macromolecule in prokaryotes are less understood (Bugg et al., 2012; Tian et al., 2014). There are examples of peroxidases from the DyP family, a few MnPs and putative LiP sequences found in bacterial lignin-degrading isolates (Tian et al., 2014; Cragg et al., 2015; Ravi et al., 2017; Bomble et al., 2017). The first evidence of the genetic repertoire associated with lignin degradation in bacteria was obtained by sequencing the genome of the actinobacteria *Amycolatopsis* sp. 75iv2 (Brown et al., 2012). Moreover, genome sequencing of the actinobacteria *Amycolatopsis* sp. 75iv2 revealed the presence of a large number of genes encoding oxidative enzymes, such as heme peroxidases, laccases, and cytochrome P450s (J. R. Davis et al., 2012). The evidence was corroborated by secretome data that revealed two abundant heme-containing proteins that are closely related to amyco1 orthologs, which were previously shown to act synergistically in degrading biomass by uncapping new phenolic sites (Brown et al., 2011).

After lignin depolymerization, the resulting mix of phenolic compounds are assimilated via specific upper funneling pathways, depending on their substitution pattern, into central aromatic intermediates (e.g. catechol, protocatechuic acid or gallic acid). There is no single natural species described so far that carries all the alternative funneling pathways and that could proliferate on any lignin-derived aromatic compound that could act as the carbon and energy source. The interested reader is referred to a number of recent comprehensive reviews on the topic (Woo et al., 2014; Abdelaziz et al., 2016; Abdelaziz et al., 2016; Xu Z. et al., 2018; Xu et al., 2019).

The primary bacterial models used for lignin degradation studies are represented by members of Proteobacteria and Actinobacteria, followed by a minor fraction performed by Firmicute species (Tian et al., 2014; Vitorino and Bessa, 2018). Among them, particular interest is focused on Actinobacteria and Proteobacteria, groups that are recognized for their metabolic diversity and biotechnological relevance (Bruce et al., 2010; Melo-Nascimento et al., 2018). Evidence across the prokaryotes indicates that the β-ketoadipate pathway is the major catabolic node for assimilating aromatic compounds, although alternative solutions also exist. Several bacterial strains have been reported to metabolize a significant number of different low-molecular-weight aromatics (Woo et al., 2014; Kanehisa et al., 2016).

*Pseudomonas spp.* have the ability to assimilate a diverse set of aromatic compounds and are often found among bacteria isolated for their ability to grow on aromatic substrates. is easy to grow in minimal medium and is genetically accessible for metabolic engineering by a comprehensive genetic toolbox [see review by Nikel and de Lorenzo (2018)]. It is therefore considered a good model organism for studying lignin biotransformation, and for developing novel industrial production strains. The *P. putida* KT2440 strain has enabled a better understanding of uptake of aromatic compounds (e.g. ferulic acid, hydroxybenzoate, benzoate, and vanillate) (Ravi et al., 2017) as well as the elucidation of the metabolic pathways that lead to production of medium-chain-length polyhydroxyalkanoates (MCL-PHAs) (Linger et al., 2014), or muconic acid, which can be further hydrogenated to the Nylon pre-polymer adipic acid (Vardon et al., 2016). Pseudomonas spp. es. For example, *Sphingomonas* (*Pseudomonas*) *paucimobilis* SYK-6, was found to be able to grow in 5,5 -dehydrodivanillic acid (DDVA), a waste product of the pulp-bleaching industry, and it could also use vanillate and syringate as a single carbon source (Nishikawa et al., 1998). *Rhodococcus erythropolis* can grow in minimal media containing a broad range of aromatic compounds such as *p*- and *m*- cresol, biphenyl, ferulic acid, vanillic acid, and veratryl alcohol (Masai et al., 1999). The *Klebsiella* sp. strain BRL6-2 revealed four putative peroxidases including glutathione and DyP-type peroxidases, and it has a full protocatechuate pathway for processing catechol degradation to β-ketoadipate, as in *Cupriavidus basilensis* OR16 and *Sphingomonas paucimobilis* SYK6 (Masai et al., 1999; Woo et al., 2014; Bugg and Rahmanpour, 2015). Currently, it is well established that heme peroxidases, such as DyPs, play a central role in the bacterial’ ligninolytic ability. Heme peroxidases including DyP (and other lignin peroxidases) are more effective than classical peroxidases for degrading aromatic compounds, which constitute 90% of the lignin (McLeod et al., 2006). The Dyp family promotes the oxidation of Mn(II), and via β-aryl ether lignin model compounds in *R. jostii*, RHA1 is one of the best Dyp metabolic roles established thus far (Ahmad et al., 2011). Moreover, The *Klebsiella* sp. strain BRL6-2 revealed four putative peroxidases including glutathione and DyP-type peroxidases, and it has a full protocatechuate pathway for processing catechol degradation to β-ketoadipate, as in *Cupriavidus basilensis* OR16 and *Sphingomonas paucimobilis* SYK6 (Masai et al., 1999; Bugg et al., 2012; Woo et al., 2014). Finally, some yeast species have been described as lignin depolymerizer microorganisms, such as *Rhodotorula graminis*WP1 and *Rhodotorula mucilaginosa* CBS17, which were able to grow on lignin-derived carbon sources such as catechol, protocatechuate, caffeic acid, vanillic acid, and others by breaking down these molecules through the β-ketoadipate pathway (Tian et al., 2014).

In addition, many ligninolytic microorganisms have already been discovered and have had their metabolic pathways described, and there is still a vast diversity of organisms to be found. Therefore, there is a current need for developing efficient screening methods that enable the identification of new microbial activities. The following sections will detail the most commonly used screening methodologies to explore the ligninolytic microbiome broadly.

## Cultivation-Based Methodologies Enable the Isolation of Lignin- Degrading Microorganisms

Culture-dependent methods for isolating lignin-degrading microorganisms have been widely used since the end of the last century (Mercer et al., 1996; Temp et al., 1998). Those techniques are well stablished and have traditionally been focused on identifying LMEs applied to the paper and pulp industry, and on determining the activity of the primary classes of ligninolytic enzymes such as laccases, peroxidases and other oxidative enzymes (Figure 2).

Cultivation-based methods enable the screening of easily cultivated microorganisms and their respective enzymes, also giving an overview of this genome characterization. Over the last decade, this focus has turned toward isolating microorganisms with direct potential for lignin valorization in a multiproduct biorefinery setting by assimilating specific TLs, DTLs or model aromatic compounds and for producing high-value chemicals. Although some of the traditional screening techniques for LMEs have become obsolete over the years, such as ^14^C autoradiography (Temp et al., 1998), other techniques such as colorimetric assays are still often used, especially when coupled with high-throughput approaches (Taylor et al., 2012; Ohta et al., 2012).

**FIGURE 3:**
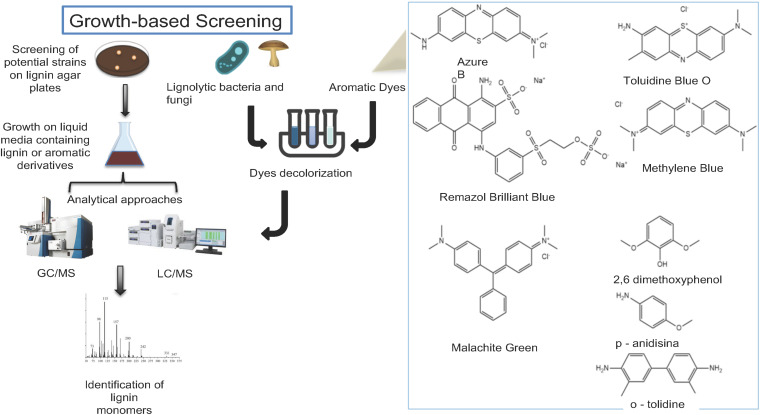
Overview of growth screening methods. Cell growth and activity-based screening methods for the identification of lignin-degrading microorganisms and enzymes. Growth in solid and liquid media containing lignin and aromatic dyes (Colorimetric assays) coupled with GC-MS, LC-MS. The structures of common substrates used in the activity-based screening assays is shown.

When qualitative and quantitative assays are implemented, important factors such as time, sensitivity cell growth requirements, and the number of samples to be screened must be considered (Motato-Vásquez et al., 2016; Sun et al., 2018). The qualitative detection of lignin-degrading bacteria and fungi are generally performed using natural substrates that resemble the lignin structure (Figure 3; Table 1). The most common practice is to use aromatic dyes such as azure B, Remazol Marine Blue, toluidine blue, methylene blue, malachite green, Remazol Brilliant Blue R and indulin AT as carbon sources in the growth medium and to monitor the modification in the color from the degradation by the oxidative enzymes produced by the microorganisms (Figure 3; Raj et al., 2007; Nozaki et al., 2008; Zhou et al., 2017; Xu et al., 2019). In liquid media, the degradation of methylene blue, toluidine blue, and malachite green dyes can be quantified by measuring the optical density of the sample at a wavelength of 620 nm using a spectrophotometer. Strains such as *Pandoraea norimbergensis*, *Pseudomonas* sp. (Bandounas et al., 2011) and *Klebsiella* sp. (Melo-Nascimento et al., 2018) were described as ligninolytic microorganisms when using this approach in media containing those three dyes. In addition, azure B dye has been previously used as an indicator of lignin peroxidase (Lip) and Mn-dependent peroxidase (MnP) activities. This methodology was successfully used to retrieve *Bacillus* sp. (Raj et al., 2007), basidiomycetes fungi (Nozaki et al., 2008), actinobacteria, *Klebsiella pneumoniae* (Xu R. et al., 2018), *Pseudomonas putida* and *Ochrobactrum tritici* presenting lignin-degrading activity in soils, leaf mold samples, and termite guts (Zhou et al., 2017).

**TABLE 1:** Comparison between different culture-dependent screening methods for detecting lignin degrading activities.

Screening method	Samples/biological model	Targeted molecule	Advantages	Disadvantages	References
Growth-based assay	Enriched vegetal compost sample *P. putida* (model organism)	vanillin, vanillate, 4- hydroxybenzoate, p-coumarate, benzoate, and ferulate.	Understand microorganisms’ metabolic pathways and uptake of aromatic compounds.	Low applicability for difficult -to- grow microorganisms.	Ravi et al., 2017
Colorimetric assay	Enriched soil samples *Pseudomonas* sp. and *Rhodococcus* sp. (model organisms)	toluidine blue, methylene blue, malachite green, Congo red.	Accurate evaluation of lignin monomers degradation.	Ligninolytic activities may vary in different types of lignin	Bandounas et al., 2011; Melo-Nascimento et al., 2018
	*Pseudomonas* sp. (MPH enzyme and promoter) and *E. coli* as host	Synthetic Organophosphates	Organophosphate-degrading enzyme identification by fluorescence	Activities found in synthetic molecules may not be the same in lignin derived molecules	Jeong et al., 2012
	Enriched soil samples	High- and low-molecular-weight Kraft lignin	Identification of new lignin-degrading strains and enzymes.	The method is restricted to samples with nitrate.	Taylor et al., 2012
	*Streptomyces violaceoruber, Streptomyces spiroverticillatus* Termite gut bacteria; *Enterobacter hormaechei* and *Bacillus licheniformis*.	Azure B	Does not require specialized expertise. Low cost.	Time-consuming, requires high amount of enzyme.	Zhou et al., 2017
LC/MS and HPLC	*Microbacterium, R. erythropolis* and *Sphingobacterium* strains	High- and low-molecular-weight Kraft lignin.	Analysis of lignin biotransformation by analytical tools.	Same m/z ratio in different compounds may give misleading results	Taylor et al., 2012
LC/MS and GC/MS	Deep-sea sediment samples	p-coumaric acid, ferulic acid, sinapic acid, vanillin, syringaldehyde, protocatechuic acid, p-hydroxybenzaldehyde, and veratryl alcohol	Analysis of lignin biotransformation by analytical tools.	Compounds with similar structures and m/z ratio may give misleading results	Ohta et al., 2012
Enzymatic assay	*Bacillus* sp., *Trichoderma* and *Actinomycetes*	ABTS	Accessible, safe, and convenient. The substrate is soluble, stable, and readily available.	Time-consuming, less sensitive, not accurate.	Alcalde et al., 2005; Vasilchenko et al., 2012; Huang et al., 2013; Monssef et al., 2016
Enzymatic assay	140 bacterial strains from the Peru rainforest	lignin dimer guaiacylglycerol-b-guaiacyl ether	Identification of lignolytic strains, by laccase interaction with ABTS.	Tests were limited to laccase activity	Huang et al., 2013
Native gel electrophoresis	*Trametes gallica, Ganoderma lucidum*, and *Streptomyces badius*	o-tolidine, guaiacol and ABTS	Rapid, accessible, secure, and can be applied for crude enzymes	Less sensitive, requires a substantial amount of enzyme, and poor gel quality and bands.	Adhi et al., 1989; X. Sun et al., 2004; Kumar et al., 2017
Chemiluminescent (CL)	Actinomycetes abundant in *Streptomyces* sp. and *Thermomonospora* sp. screened for peroxidase	luminol	Sensitive, and rapid. Successfully applied to the crude sample.	Weak signal, short luminescence time, sensitive to external factors	Mercer et al., 1996; Zhang et al., 2018
Capillary electrophoresis	Culture filtrate of Phanerochaete chrysosporium	malonate and veratryl alcohol	Sensitive, different enzyme activities measured without purification.	Protein adsorption to capillary walls. Small, injectable sample size (1–30 nL).	Kudo et al., 2017
^14^CO_2_ autoradiography	*Thermomonospora mesophila, Streptomyces* sp., *Pycnoporus cinnabarinus*, and *Ceriporiopsis subvermispora*.	^14^C-ring-labeled dehydrogenation polymer (DHP) (synthetic lignin)	Accurate, sensitive, screening of a large number of samples.	Laborious, time-consuming, requires expertise, sensitive to culture conditions.	Temp et al., 1998
Spectrophotometric assays associated with nitrated lignin	*S. viridosporus, Nocardia autotrophica, Rhodococcus sp*.; *P. putida* and *Rhodococcus* RHA1 (positive controls) *Bacillus subtilis* and *Leuconostoc mesenteroides* (negative controls)	nitrated milled wood lignin	A semiquantitative and scalable method. Multiwell microtiter plates assay.	Growth-dependent. Nitrated lignin assays are time-consuming.	Ahmad et al., 2011

Chromogenic substrates are also used to screen for lignin-degrading microorganisms. The types of substrates as well as the enzymatic activity related to them are summarized in Table 1. Their advantage in comparison to dyes is the possibility of quantifying the enzyme activity and of developing high-throughput screening assays (Vasilchenko et al., 2012; Chong et al., 2018; Xu et al., 2019). The Prussian blue assay is an example of a colorimetric assay used for addressing lignin-degrading bacteria such as *Rhodococcus pyridinivorans* and *Rhodococcus opacus* (Vasilchenko et al., 2012; Xu et al., 2019). Furthermore, the Prussian blue assay has also been used to isolate cellobiose dehydrogenases (CDHs) from the ascomycetes *Diplodia pinea, Melanocarpus albomyces, Papulaspora biformospora*, and *Chaetomium* sp. (Vasilchenko et al., 2012; Xu et al., 2019). 2,2’-and-bis (3-ethylbenzene-thiazoline-6-sulfonic acid)-ABTS is widely used for laccase detection once this nonphenolic substrate releases a green color after enzyme oxidation. A chromogenic ABTS assay coupled to analytical techniques was used to isolate laccases from different microorganisms such as *Bacillus pumilus, Bacillus atrophaeus* (Huang et al., 2013), *K. pneumoniae, P. putida*, and *Ochrobactrum tritici* (Xu Z. et al., 2018). The color change is generally monitored in 96-well microtiter plates at an absorbance of 420 nm. After that, selected enzymes are characterized by mass spectrometry. For fungal laccases, those produced by *Pycnoporus cinnabarinus* (Camarero et al., 2012) and *Pycnoporus sanguineus* use ABTS as the substrate (Alcalde et al., 2005; Huang et al., 2013; Xu et al., 2019).

For peroxidase isolation, one efficient method is to use luminol as a chemiluminescent substrate. Chemiluminescence is a rapid and sensitive technique used to screen for peroxidase activity using enzymes such as anionic peroxidases and horseradish peroxidase (HRP) in a small group of untreated culture supernatants. *Streptomyces* species were screened for peroxidase activity using the chemiluminescent assay (Mercer et al., 1996). The hydrolases-luminol system produces an excited state intermediate in the presence of H_2_O_2_, which can be detected in immunoassays. Although a disadvantage of this method is that this assay has a short luminescence time, imposing a need for enhancers such as substituted phenols and boronic acids, for example, they also require the use of magnetic beads or nanoparticles shown to be crucial for obtaining a more stable and robust fluorescent signal (Zhang et al., 2018).

The direct activity measurement of enzymes separated on electrophoresis gels is another approach to detecting active lignin enzymes in culture supernatants or in crude extracts isolated after microbial growth on specific substrates. Native SDS-PAGE gels soaked or copolymerized with substrates such as guaiacol, ferulic acid ABTS, *O*-dianisidine, and *O*-toluidine have been used for laccase and peroxidase detection. Positive activity is detected by the formation of a colored product (Achar et al., 2014; Kumar et al., 2017). Other substrates such as 2,6-dimethoxyphenol and *p*-anisidine were used to detect the laccase activity (Sun et al., 2004; Tomani, 2010; Kumar et al., 2017). The phenol peroxidase activity could instead be detected using DOPA or amino-antipyrine and H_2_O_2_ as substrates (Adhi et al., 1989). The peroxidases are stained red in both protocols. Gel electrophoresis offers the advantage of a direct and quick measurement of relatively pure enzymes. However, poor gel quality and band resolution may still preclude the conclusions. An alternative to native PAGE, in which the functional properties of enzymes are also maintained, is to use capillary electrophoresis (CE) to separate the complex protein mixtures, coupled with an activity assay. This method was used to screen for peroxidases such as manganese peroxidase (MnP) and lignin peroxidase (LiP) simultaneously in a capillary reaction using the fungus *Phanerochaete chrysosporium*as the enzyme source (Kudo et al., 2017).

Guaiacylglycerol-β-O-4-guaiacyl ether is a phenolic substrate with high β-O-4-linkage content, with a structure very similar to that of lignin. Therefore, it was used as a substrate to screen the termite gut microbiome, yielding a strain of *Trabulsiella* sp. as the highest substrate consumer (Suman et al., 2016). The same approach was used to identify strains of *Bacillus* sp. from rainforest soils that presented lignin-degrading activity (Huang et al., 2013). The use of GGCE as a substrate enabled the identification of glutathione S-transferases and NAD-dependent dehydrogenases from *S. paucimobilis* (Masai et al., 1999) as well as NAD-dependent dehydrogenases, glutathione S-transferase (GST) and a GSH-dependent lyase from *Sphingobium* sp. acting on the cleavage of the β-O-4-linkage (Pereira et al., 2016). In both studies, the breakdown of β-O-4-linkages was either measured by gas chromatography coupled to a mass spectrometer (GC-MS) or a liquid chromatographic apparatus coupled to a mass spectrometer (LC-MS) (Masai et al., 1999; Pereira et al., 2016).

The biotransformation of lignin can also be measured by nitrated lignin assays coupled to analytical tools such as LC-MS and GC-MS (Ahmad et al., 2011; Taylor et al., 2012). This assay consists in spraying inoculated agar plates with a solution of nitrated lignin, which is prepared using MWL together with acetic and nitric acid. When a strain can degrade lignin, a fluorescent yellow signal is released, which can be measured at an absorbance of 430 nm. In a previous study, *P. putida* and *R. jostii RHA1* were identified by this method (Ahmad et al., 2011). Twelve other ligninolytic strains were isolated from a metagenomic enriched soil sample containing MWL. Those strains were capable of growing on M9 minimal media containing high- and low-molecular-weight lignin that was also treated with nitrated lignin solution (Taylor et al., 2012). Lignin biotransformation by selected strains was evaluated by coupled LC-MS and GC-MS, producing mostly vanillin, oxalic acid, and protocatechuic acid as detectable products, which implied the presence of extracellular peroxidase activity (Table 1). As an alternative quantitative approach, a mass spectrometry-based enzymatic assay approach called the NIMS enzymatic (Nimzyme) assay, which lacks the previous fractionation step, is under development (Deng et al., 2018).

Based on a system that immobilized substrates containing β-O-4 linkages (Northen et al., 2008; Deng et al., 2018), the designed β-aryl ether bond-containing model uses lignin dimer substrates for studying the activities of LMEs. The activities of manganese peroxidase (MnP) from *Nematoloma frowardii* and laccase from *Trametes versicolor* were tested, showing the liberation of products that arise from the cleavage of the carbon-carbon single bond and oxidative reactions. Therefore, mass spectrometry-based enzymatic assays are robust and promising approaches to a comprehensive understanding of the enzymatic cleavage of β-aryl ether (β-ether)-linkages and the identification of novel enzymatic activities responsible for lignin breakdown, and different compounds based on the mass-to-charge ratio of these compounds (Deng et al., 2018).

As exemplified above, there is a vast quantity of assays that may be applied to culture collections varying in sensitivity and detection limit. Those are frequently utilized to scan the ligninolytic potential of a given microorganism, sample or collection. Table 1 summarizes the available methodologies as well its main advantages and disadvantages.

## Utilization of Genome Databases for Isolating Genes Involved in Lignin Degradation

It has been estimated that only 1–10% of the existing microbial biodiversity has been explored due to the technical challenges of cultivating microorganisms in a laboratory environment. Therefore, DNA-based methods represent an alternative to overcome cultivation problems. (Zhu et al., 2014; Vitorino and Bessa, 2018). Therefore DNA-based methods enable the screening of lignolitycal activity by searching for genes encoding key enzymes for lignin biotranformation.

**FIGURE 4:**
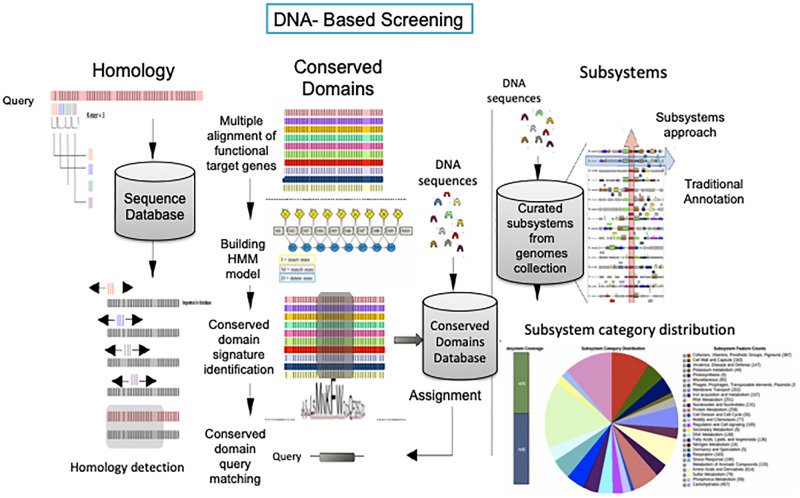
DNA-based annotation strategies. Homology-based annotation. The BLAST search algorithm uses 3 k-mer words to anchor and extend the alignment for establishing homology between the queried sequence and the deposited sequence. Conserved domain-based annotation. Hidden Markov Models (HMM) are built based on a multiple alignment from homologous sequences resulting in conserved domain signatures for specific family proteins. The signatures are used as *in silico* probes against DNA sequences (or vice versa). Subsystem-based annotation. DNA sequences are allocated in curated subsystems (experimental data) based on K-mer searching for the identification of isofunctional homolog genes in closely related genomes harbored in protein families assigned by the Fellowship Interpretation of Genomes (FigFam), connecting the functional role and in chromosomal cluster with genes implementing functional roles from the same subsystem (red arrow along the genomes). The pie chart (below) and the genomic arrangements (on the right) are a graphic representation of the SEED server subsystem distribution category for *Pseudomonas putida* KT2440.

The sequencing data have revealed that the microbial diversity was much more extensive than believed before (Hughes et al., 2001; Curtis and Sloan, 2004). The number of gene coding sequences associated with the extracellular activities involved with lignin degradation has increased exponentially in the last 20 years (Figure 4). Thus, the expansion of databases represents a valuable opportunity for mining new genes involved with the intracellular steps for ring structure conversion present in microbes that are able to degrade aromatic compounds (Picart et al., 2015). Traditional databases such as NCBI and PATRIC are widely used for gene/function bioprospecting (Davis et al., 2012; Sayers et al., 2020). Most of the data could be downloaded for stand-alone applications. However, they are usually remotely accessed by the user and present a variety of dataset in different formats (for instance, nucleotide and amino acid sequences in fasta and GenBank format) and provide an interesting variety of bioinformatic tools which allow comparison, identification, and characterization of genes and genomes. An efficient process of gene annotation is fundamental for the identification of functional genes and the establishment of phylogenetic analysis and comprehensive studies of the influence of the genetic variation in an enzyme activity, which is a fundamental knowledge to drive the choice of candidate genes for genetic engineering.

In recent years, tools aimed at the whole genome-based metabolic reconstruction, metabolic interactions networks, and flux balance analysis have become increasingly available through the virtual environments that harbor the primary databases. The recently published eLignin database has gathered a large amount of published literature on the catabolism of aromatic compounds and has made it available in a simple Internet-based software tool that can search for microorganisms, enzymes, pathways, and metabolites of interest (Brink et al., 2019). Other sources of information include the Kyoto Encyclopedia of Genes and Genomes (KEGG) and other protein databases, such as UniProt, ExPASy and Pfam, that will be further mentioned in this review (Artimo et al., 2012; Kanehisa et al., 2016, 2014). Although the lignin degradation system is best described for fungi, the increase in databases as a function of the number of cured sequences deposited in databases has increasingly allowed the characterization of genes involved in the lignin conversion processes. Among the well-established lignin-degrading bacteria, members from Actinobacteria and Proteobacteria represent relevant study models for genome-based analysis. Thus, it has been possible to identify and characterize targets for the manipulation of metabolic routes to establish the variety of strategies used by microorganisms in the degradation processes of lignin and its fragments, as described below.

*Rhodococcus jostii* RHA1, has been described to depolymerize Kraft lignin into oxalic acid and protocatechuic acid (Taylor et al., 2012). Available in the NCBI, the *R. jostii* RHA1 is one of the largest known bacterial genomes (approx. 9 Mb) and contains 203 genes coding for oxygenases (Masai et al., 1999; Bugg et al., 2012; Woo et al., 2014). The *R. jostii* RHA1 genome sequencing revealed the presence of twenty-six peripheral pathways and eight central pathways are involved in the catabolism of aromatic compounds, including modifications by monooxygenases and dioxygenases. The biochemical characterization of the previous putative genes of RHA1 for DyPs showed peroxidase activities, suggesting their implication in lignin degradation. Members of the Dyp peroxidase family were annotated as DypA and DypB, on the basis of bioinformatic analysis in the genome of *R. jostii* RHA1 (Ahmad et al., 2011). Initially, a structure-based alignment of DyP sequences deposited in Protein Data Bank (Berman et al., 2000) was used as a profile to align the DyP sequences deposited in the Peroxibase database (Koua et al., 2009). The final alignment was used to determine similarity among the queries and reference sequences based on phylogenetic analysis. The DyPs were also found in Pseudomonas strains and showed oxidation activity for Mn(II). Two well-studied and shared proteins (DyPPa and TyrA) that are homologous to PmDyP in the primary structure were investigated and the gene encoding pmDyP of *Pseudomonas* sp. Q18 was amplified using primers, designed based on the gene sequence of the hypothetical DyP-type peroxidase of *Pseudomonas* sp. JY-Q (CP011525.1) (analyzed via the ExPASy server tools). The gene of PmDyP was cloned and expressed, According to results, PmDyP presented the ability to break down alkaline lignin and native lignocellulosic material. Compared with wheat straw and corn stalk, the treatment of switchgrass by *Pseudomonas* sp. Q18 showed the highest weight loss of dry biomass, almost 25% (Yang et al., 2018). Pseudomonas strains are recognized as potential lignin degraders. The genome draft of *Pseudomonas* sp. strain YS-1p revealed that it contains genes that code for enzymes needed for lignin and lignin-derived aromatic compound degradation including laccase, DyP-peroxidase, β-etherase, vanillate O-demethylase, a feruloyl esterase, carboxylesterase, cytochrome P450, and chloroperoxidase (Prabhakaran et al., 2015). All proteins sequences were functionally annotated using a combination of NCBI Blast and HMMER against the PFAM database.

Some pseudomonas genomes are widely bioprospected for genes involved with aromatic structures, which could be helpful for lignin degradation biotechnological applications. Genome based analysis allowed the use of the catechol metabolic node as a target for genetic engineering in *Pseudomonas putida* KT2440 for the production of muconic acid from catechol and upstream aromatics. At the core of the cell factories created was a designed synthetic pathway module, comprising both native catechol 1,2-dioxygenases, catA and catA2, under the control of the Pcat promoter (Kohlstedt et al., 2018). The generated library of synthetic promoters opens various application-based possibilities for the fine-tuning of gene expression in *P. putida* KT2440 and related strains, such as muconic acid producers to provide first nylon from lignin in a cascaded chemical and biochemical process.

Regarding laccase-like activities, bioinformatic analysis revealed that copA gene was found in the genomes of bacterial strains capable of lignin oxidation. The function of CopA has been previously studied in Pseudomonas syringae in the context of copper resistance. However, a double gene deletion of copA-I and copA-II genes in *P. putida* KT2440 was constructed, and this mutant showed diminished growth capability on different small aromatic compounds related with lignin degradation (Granja-Travez and Bugg, 2018). The genes were found by a search in the online database UniProt, using the amino acid sequence of CopA-II (UniProt code Q88C03) as a probe for the BLAST search. The study suggests some accessory role in lignin oxidation by CopA in the presence of Cu(II) ions.

Although most studies involving lignin and lignin-derived aromatic degradation have been mostly focused on isolation or culture-independent methods, the identification of coding genes for LME in degrading systems has also been successfully performed using a metagenomic approach. Ligninolytic consortium analyses can reveal the novel genomes and pathways involved in lignin modification and valorization. Soils are commonly used as a microbial source for enrichment processes due to their high complexity and metabolic versatility associated with the microbial communities adapted to a variety of carbon and energy sources. An agriculture soil used to grow sugarcane presented nearly 3% genes related to peroxidases, dye-decolorizing peroxidases, and laccase domains belonging predominantly to the Actinobacteria and Proteobacteria (Moraes et al., 2018). The glutathione-dependent β-etherases catalyze the reductive cleavage of β-O-4 aryl-ether, and their presence is indicative of the ability to access technical lignin. A CAZyme functional assignment allowed for the identification of enzyme families with AA associated with microbial consortia developed by the enrichment of soil in climbing vines, grasses, and corn straw, allowing for the identification of a microbial consortium (Lima et al., 2016). The results showed that the most abundant AA families in the consortia were AA6 (1,4-benzoquinone reductases) and AA10 (LPMOs), followed by the low-abundance families AA2 (lignin peroxidases), AA7 (*gluco-oligosaccharide oxidases*) and AA4 (*vanillyl-alcohol oxidases*). The characterization of AA10 enzyme activities suggests a model for enzymatic cellulose depolymerization based on the oxidative cleavage of endo glycosidic bonds in crystalline cellulose, creating new chain ends that can be accessed by cellobiohydrolases (Horn et al., 2012). Moreover, the abundance of the AA6 family also suggests an intracellular activity involved in the biodegradation of aromatic compounds. However, there is limited information regarding the actual role of these proteins in a lignocellulolytic bacteria-dominated consortium.

**FIGURE 5:**
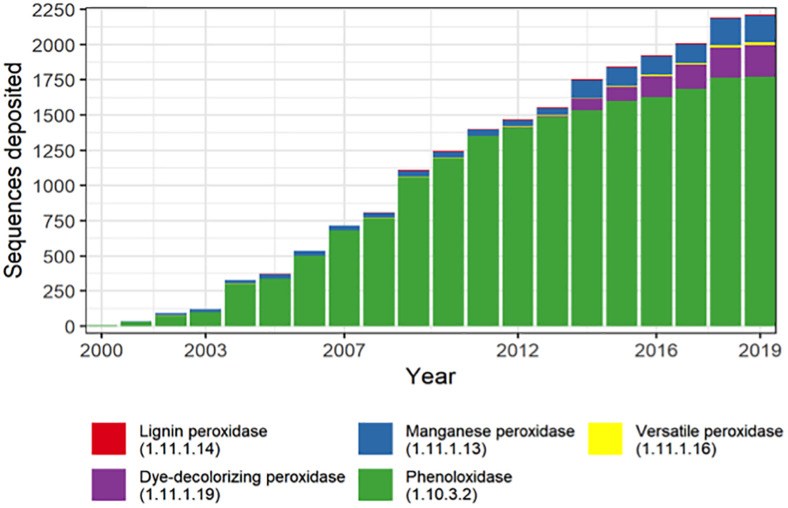
Lignin depolymerization-related genes annotated over the last 20 years.

Currently, a variety of annotation tools are available for the identification and characterization of the genetic repertoire involved in lignin degradation (Figure 5). The approaches discussed below offer the opportunity to explore the molecular functional diversity at different levels of complexity, including individual (homology), protein families (conserved domains) and metabolic context subsystems. A more accurate annotation of misrepresented or distantly related genes is successfully reached by using specialized databases and combining different annotation strategies that consider the genomic context and functional features of a specific protein family. Frequently, the choice of the most appropriate annotation strategy is determined by the goal of the study.

## Strategies for Searching Genes From Databases

Homology-based annotation is one of the most popular strategies, and it is based on the similarity degree among homologous gene sequences. The similarity inference is determined by the alignment (pairwise or multiple alignment) of the sequences. This strategy remains one of the most frequently used for the annotation of single microbial genes, genomes, and metagenomes.

The most popular search tool at the NCBI is BLAST (Basic Local Alignment Search Tool) (Altschul et al., 1990; Pevsner, 2009). The BLAST tool has been extensively used to identify genes based on the homologies used for genome annotations of lignin-degrading prokaryotes through the NCBI PGPA (Tatusova et al., 2016). The PGPA is used as the initial search for homologs in the nonredundant NCBI database. However, the NCBI is not a specialized database for lignin degradation. Nevertheless, homology-based annotation efforts are based on incomplete functional annotations. Annotated genomes typically contain 30–50% genes without functional annotation, resulting in missing functional annotations in newly sequenced genomes.

In contrast, the conserved domain-based annotation is performed by identifying conserved regions through the global alignment of sequences within a particular protein family. The conserved domains do not necessarily represent the catalytic domain of an enzyme; however, by associating the domains with specific protein families, this annotation strategy directly associates the sequence with the list of functions performed by the members of that family. Because protein sequences are often more conserved over evolution than nucleotide sequences, the search for conserved domains is more efficient than the simple search for a sequence similarity for the identification of new genes. Among the advantages of the conserved domain-based annotation is the ability to improve the identification of new sequences, it is distantly related to those deposited in databases. In general, the sequences presenting at least 40% identity with 70% coverage in comparison with the deposited sequences are considered homologous, according to empirical evidence (Eddy, 2009; Hinz and The UniProt Consortium, 2010). In cases in which the similarity is close to the mentioned limit, the homology may not be identified by the algorithms used by BLAST search strategies.

The conserved domain-based annotation is broadly used to identify biomass-degrading activities. Although most of the studies that address the degradation of lignocellulosic biomass focus on cellulolytic and hemicellulolytic enzymes, they have contributed to the expansion of knowledge about the genetic repertoire involved in lignin degradation. In general, the enzymes involved in the cleavage of complex carbohydrates and the control of carbohydrate metabolism are called CAZymes (Lombard et al., 2014). The emergence and popularity of CAZymes (carbohydrate-active EnZymes) has resulted in a web server for automated carbohydrate-active enzyme annotation (Huang et al., 2018). The enzymes that make up the CAZymes are classified based on the similarity of sequences and protein structures and according to the CAZy database^2^. Currently, there are six primary classes in the CAZy database, which are known as glycosyl hydrolases, carbohydrate esterases, glycosyltransferases, polysaccharide lyases, carbohydrate-binding modules and AAs. The AAs are represented by redox type enzymes that act in conjunction with other CAZymes in the degradation of the plant cell wall (Cantarel et al., 2009; Quinlan et al., 2011; Li et al., 2012; Bey et al., 2013). Described initially as cellulases, the GH61 enzymes were reclassified as AA due to their copper-dependent lytic polysaccharide monooxygenase (LPMO) activity (Quinlan et al., 2011; Li et al., 2012). The LPMO catalyzes the oxidative cleavage of cellulose using low-molecular-weight reducing agents such as ascorbate, gallate, reduced glutathione, and even lignin (Bey et al., 2013). Thus, the AA class improved and extended a former classification dedicated to ligninolytic enzymes (Levasseur et al., 2013).

While the conserved domain-based annotation represents a critical tool to identify activities for lignocellulosic biomass reconstruction, it cannot place those activities in a metabolic context, which makes the reconstruction of metabolic pathways involved with lignin degradation difficult. Otherwise, annotation strategies known as Rapid Annotation using Subsystem Technology and gene orthology can place the identified genes in a metabolic context. The subsystem can be understood as a set of functional roles that implement a particular biological or structural process (Gerlt and Babbitt, 2001). The subsystem spreadsheet is populated with all the genomes that have functional roles associated with the metabolic pathways for a specific subsystem. The proteins that make up the subsystems form families of isofunctional homologs aggregated into subsystems. Each subsystem is curated by a group of scientists specializing in specific metabolic pathways, establishing which genes are involved and uncovering their genomic architecture (FIGfam-Fellowship Interpretation of Genomes Family). The SEED server is the most popular platform available to perform RAST, offering an efficient and automatized annotation workflow (Aziz et al., 2008; Hu et al., 2018). Sequences are uploaded and iteratively flow through the gene calling and are validated by k-mers. If a gene candidate has not been assigned a subsystem-based functional role, and it has flanking genes with subsystem-based functional roles, then it is compared with the nearest neighbors. This strategy allows users to compare the genomic neighborhood of a given gene across genomes, providing a powerful means for finding and correcting gene calls and for predicting new functions based on conserved genomic context. For example, metabolic pathways for aromatic compound degradation can be used to track microbial ligninolytic potential. The aromatic compound subsystems harbor well-established pathways involved in the degradation of complex organic molecules (i.e. pesticides, dyes, and hydrocarbonate), which are also shared by the lignin degradation metabolism, primarily the steps associated with lignin fragment degradation. The peripheral pathway of the metabolism of aromatic compounds is composed of three subsystems, the quinate degradation subsystem, benzoate degradation, and 4-hydroxybenzoate degradation. Thus, subsystem-based annotation strategies represent a promising tool for the identification, characterization and metabolic reconstruction of new ligninolytic prokaryotes (Tian et al., 2014), but it is still necessary to increase the representativity of ligninolytic microbes toward curating the complete lignin degradation subsystems, including the first (extracellular) and second stage (intracellular) of lignin molecule deconstruction. Moreover, the RAST server provides programmatic remote access to the Model SEED biochemical and genome-scale metabolic model database, integrating all the reactions and compounds found in the Kyoto Encyclopedia of genes and Genomes (KEGG) database of published genome-scale metabolic models into a single, nonredundant set (Kanehisa et al., 2016, 2014). In connecting the gene orthology with EC numbers, the algorithm can connect them in functional blocks to build metabolic networks in a variety of hierarchical levels, delivering a comprehensive representation of the role of the genes in a cellular metabolic context.

The efficient producer of peroxidases for lignin modification identified as *Pseudonocardia autotrophica* Strain DSM 43083 (Grumaz et al., 2017) was annotated using PROKKA software. For gene finding and translation, PROKKA performs homology searching via BLAST and HMMER against a set of public databases (CDD, PFAM, and TIGRFAM) (Seemann, 2014). The *in silico* search for oxidoreductases, which are related to the degradation of aromatic compounds and lignin, resulted in a set of genes with at least five relevant dioxygenases, three monooxygenases, and two DyP-type peroxidases. Furthermore, several putative monooxygenases are expected to hydroxylate salicylate, phenol, and p-hydroxybenzoate. *Streptomyces viridosporus* strain T7A was annotated by combining searches against the NCBI non-redundant database, UniProt, TIGRFam, Pfam, Priam, KEGG, COG, and InterPro to identify the genes encoding putative lignin-degrading enzymes, such as heme peroxidases, DyP-type peroxidases, and catalases, which are harbored by pathways for the catabolism of lignin-derived aromatic compounds (Jennifer R. Davis et al., 2013).

## Designing Biosensors to Detect Lignin Derivative Molecules

In addition to cultivation and sequence-based methods, the increase in novel screening methodologies, such as the use of biological circuits to enable the identification of novel ligninolytic activities from environmental samples, is also overcoming the difficulties involved in cultivating some strains (Vitorino and Bessa, 2018).

**FIGURE 6:**
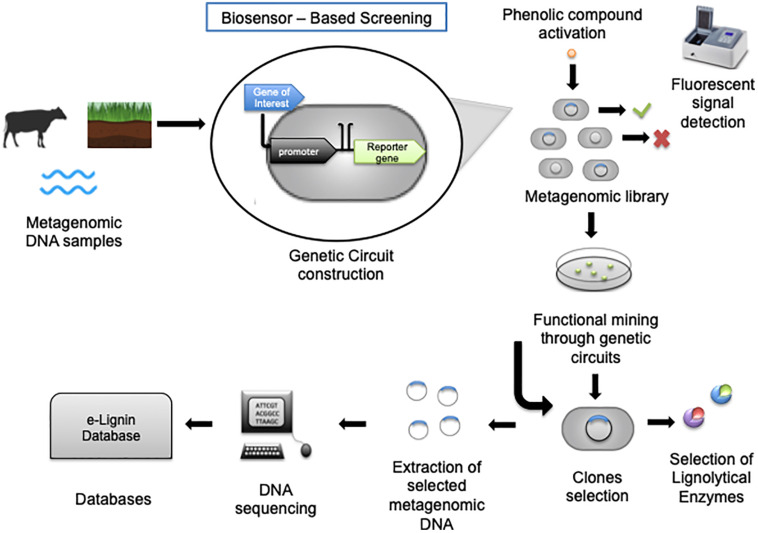
Biosensor-based screening methods. Enables the screening of metagenomic libraries, for selecting enzymes for industrial purposes and DNA sequencing. The method begins with selecting metagenomic DNA from environmental samples, which will be used in the genetic circuit construction consisting of the gene of interest, a strong promoter and a reporter gene. After the metagenomic library is constructed, the clones can be sorted by fluorescent signal detection through activation by a phenolic compound. Then, the fluorescent clones containing genes of interest are selected by functional mining, and lignolytic enzymes can be identified. Another option is to extract the selected metagenomic DNA, which will be sequenced and matched in databases, the eLignin database for instance, with a search for homologous lignolytic activities in the metagenomic samples.

Whole-cell biosensors consist of genetically engineered cells that are able to identify targeted compounds, and thus they can be applied to detect important aromatic lignin derivatives. The power of biosensors at measuring extra- and intracellular metabolites during bioprocess development have generally been well supported, and a significant number of reviews covering the plethora of available systems have been written (Schmid and Neubauer, 2010; F. Zhang and Keasling, 2011; Fritzsch et al., 2012; Eggeling et al., 2015; Rinaldi et al., 2016). Among those novel screening techniques, functional metagenomics in combination with synthetic biology tools enable the development of new biological circuits such as riboswitches, transcription factors, or protein-based sensors that respond to a specific stimulus. Some of those strategies have, for example, been used to identify genes encoding enzymes for lignin degradation directly from environmental samples (Helm et al., 2018). Herein, a review of recent developments in biosensors relevant to lignin biorefinery research is provided, with a focus on transcription-factor-based systems, which is most common. For information about the fundamental design principles and critical concepts of transcription factor-based reporter systems for analyte quantification, please see previous review (Mannan et al., 2017). Generally, a biosensor consists of a host cell containing a plasmid, which contains an inducible promoter and an exogenous gene of interest, which in most cases is obtained through environmental samples (Uchiyama and Miyazaki, 2013). This inducible promoter is triggered by an inducer molecule resulting in the expression of a reporter gene, such as GFP (Figure 6; Uchiyama and Watanabe, 2008; Fiorentino et al., 2009; Santos et al., 2016; Ho et al., 2018). The first step in constructing biosensors consists of selecting the targeted genes. In this context, the genes of interest code for ligninolytic enzymes, which can degrade specific phenolic compounds. Second, it is necessary to select an adequate plasmid and inducible promoter to assure the construction of competent engineered biosensor cells. Last, after the construction of the genomic library, the response to a specific inducer compound is evaluated, mostly by measuring the increase in the fluorescent signal generated by the reporter gene. Through these steps, it is possible to select the most efficient clones to detect a variety of desired molecules. Most of the advantages of whole-cell biosensors relate to their high selectivity and sensitivity to electrochemical changes, demonstrating their great applicability to environmental surveys. In addition, they enable the *in situ* identification of the compounds, because they are highly selective (Bousse, 1995; Gui et al., 2017). These approaches could evaluate how lignin derivatives are used as carbon sources by different microorganisms, even when they were not cultivated as summarized in Table 2.

**TABLE 2:** Primary biosensor-based screening methods for the isolation of lignin derivative aromatic compounds.

Screening method	Samples/Biological model	Targeted molecule	Advantages	Disadvantages	References
Enzyme Biosensor: Genetic Enzyme Screening System (GESS)	Metagenomic samples (ocean tidal flat sediments)	Phenol and cresol	Sensitive and quantitative. Detects low levels of phenol *in vivo*.	Only measures intracellular concentrations of compounds.	Choi et al., 2014
Aromatic Compounds Biosensor: Substrate Induced Gene Expression (SIGEX)	Metagenomic libraries: Groundwater contaminated with crude oil.	Salicylate, 3-methyl catechol, 4-chlorocatechol and chlorohydroquinone.	Enables the screening of targeted genes.	Most transcription regulator functions are still unknown. Only measures intracellular concentrations.	Uchiyama et al., 2005; Uchiyama and Watanabe, 2008; Uchiyama and Miyazaki, 2013
Aromatic Compounds Biosensor	*Bacillus subtilis* (transcriptional regulator PadR). *E. coli* and *Corynebacterium glutamicum* (host cells). Yeast (p-coumaric acid producer).	P-coumaric acid	Enables the biosensing of extracellular products. Broad range of applications and direct scale-up.	Limited accuracy and sensitivity due to single time measurements.	Siedler et al., 2017
Aromatic Compounds Biosensor	Environmental DNA from coalbeds	Vanillin and syringaldehyde	Very selective for syringaldehyde and vanillin	The detection of syringaldehyde by the developed biosensor was not further explored	Ho et al., 2018
Vanillin inducible promoter – Biosensor	*E. coli* (model organism)	Vanillin	Screening of lignin-degrading enzymes related to vanillin pathways.	The responsive range is limited to sublethal concentration of vanillin.	Sana et al., 2017

Genetic circuits using transcription regulators from *Pseudomonas* sp. in combination with a highly expressed GFP gene were constructed to detect a variety of aromatic compounds at up to 100 μM concentrations. The biosensor with transcription factor NahR (naphthalene catabolic pathway) was able to detect mostly salicylate; XylS (benzoate catabolic pathway) was also capable of detecting salicylate, in addition to benzoate; HbpR (2-hydroxyphenyl catabolic pathway) showed sensitivity to HbpR, specifically to 2-hydroxyphenyl and 2-aminobiphenyl, and finally, the dmpR (phenol catabolic pathway) that was activated by phenol (Xue et al., 2014). An additional aromatic biosensor was built from *E. coli* BL21DE3 (RIL), which was controlled by an archaeal dehydrogenase-inducible promoter from *Sulfolobus solfataricus* (Fiorentino et al., 2009).

Substrate-Induced Gene Expression enables the identification of targeted genes by inducing them with specific substrates. *E. coli* JM109 host cells were used for metagenomic library construction containing the plasmid p18GFP (Uchiyama et al., 2005). The induction system targeted transcription factors responsive to aromatic compounds; it was coupled with detection by high throughput screening with FACS. Using 3-methylcatechol, 4-chlorocatechol and chlorohydroquinone as inducers for selected genes, it was possible to isolate 12 clones containing open reading frames (ORFs) that encoded for specific transcriptional regulators that responded to these aromatic compounds (Uchiyama et al., 2005; Uchiyama and Watanabe, 2008; Uchiyama and Miyazaki, 2013).

Another similar biosensor is the GESS, which is based on circuits built for the detection of enzymatic activities such as hydrolases, alkaline phosphatases, lyases and cellulases known for degrading phenolic compounds. A metagenomic library was constructed using *E.coli* DH10B cells, which contained a fosmid with the *R. eutropha* E2 phenol-degrading operon and the transcriptional activator BenR. The presence of intracellular phenolic compounds was detected by a DmpR regulator protein that can sense aromatic compounds such as 3-hydroxyphenol, 3-ethylphenol, 4-nitrophenol, 2- hydroxybenzoic acid and give off a fluorescent signal. This screening method combined with DNA sequencing was used to identify a novel phosphatase gene with homology to an alkaline phosphatase originating from *Sphingomonas* sp. (Choi et al., 2014). Phosphatases have an important role in the posttranslational modifications of lignolytic enzymes (Rothschild et al., 1999).

Although these circuits have a broad range of applications and high performance for use in aromatic compound screening, they present some limitations. One of them is that these systems can detect only the intracellular concentrations of phenolic derivatives. Most microorganismal products used for industrial applications are secreted by the cells. Therefore, novel circuits that can detect extracellular compounds are necessary. This goal may be achieved using microdroplet technology (Becker and Gärtner, 2012).

A successful example is the measurement of *p*-coumaric acid production by *Lactococcus lactis* by co-culturing *E. coli* biosensing cells in microfluidic droplets (Siedler et al., 2017). The *E.coli* biosensor carried an EcPadR plasmid vector with the *B. subtilis* transcriptional regulator PadR, and it showed a 130-fold response in the presence of secreted p-coumaric acid by the bacterium. *p*-Coumaric acid is categorized with ferulic acid as one of the primary substrates for protocatechuic acid production, and it is an aromatic lignin derivative with important pharmaceutical properties (Linger et al., 2014; Kakkar and Bais, 2014; Ravi et al., 2017).

Some genetic circuits for vanillin detection based on transcription factors and the GFP reporter gene were also described. Vanillin is a significant lignin byproduct used as a flavoring agent, although it can cause inhibitory effects on cell growth during industrial processes (Santos et al., 2016; Ho et al., 2018). A biosensor based on a Michaelis-Menten mathematical model was constructed using *E. coli* EPI300 and an EmrRAB promoter with a GFP fluorescent output. The biosensor containing the pET15b fosmid vector exhibited selectivity to vanillin and syringaldehyde, which is a lignin monomer with a structure similar to that of vanillin, not manifesting any fluorescence in the presence of the other 36 aromatic compounds. The fluorescence output was 1,5-fold higher than that of the negative control (empty fosmid), showing a correlation between the vanillin concentration and fluorescence up to 640 μM (Ho et al., 2018). The vanillin-inducible promoter yeiW, which is native to *E. coli*, was identified by using an RNA-seq strategy for transcriptomic analysis when cells were grown in the presence of a sublethal concentration of vanillin. The yeiW promoter was used to build a GFP-based system similar to the one described above and was found to give a concentration-dependent response over a range of 0.2–5 mM vanillin. Furthermore, the output signal was shown to be specific to vanillin, and the sensor did not show a response to polymeric lignin or guaiacol, benzaldehyde, or veratraldehyde, which could also potentially be present in DTL (Sana et al., 2017). Another example of a recently developed vanillin biosensor was engineered with a QacR transcriptional regulator, from the TetR repressor family, which confers resistance to quaternary anionic compounds (Santos et al., 2016). Two *E. coli* DH5αZ1 mutants containing qacR showed higher fluorescence with an increase in tetracycline (up to 8 ng/mL) and vanillin (1 μM) concentrations.

These promising screening methods based on biosensors are expected to facilitate the identification of aromatic molecules and novel enzymes that could not be discovered previously using conventional screening methods (Choi et al., 2014; Table 2). However, growth-based and DNA sequencing methods are still widely used and present satisfactory results when screening easily cultivated strains (Ravi et al., 2017).

## Concluding Remarks

The cellulosic and hemicellulosic fractions of biomass residues have already been extensively explored over the years for the manufacture of a variety of bio-based chemicals and biofuels. Currently, R&D efforts are focused on using lignin as a substrate for producing high value-added chemicals, such as vanillin and biopolymers including Nylon 6.6, PHA and PHB (Linger et al., 2014; Sun et al., 2018). Thus, this approach promotes the full exploitation of biomass by using the existing infrastructure of biorefineries.

After lignin extraction, strategies for lignin depolymerization have to be further studied. Among the described methods, enzymatic depolymerization will receive special attention, once it is widely applicable, and due to a variety of existing ligninolytic microorganisms. Many fungal genera such as *Pleurotus* sp. and *Pycnoporus* sp. are widely known to produce oxidative enzymes such as laccases and peroxidases, which are the primary enzymes to act in lignin deconstruction (Guillén et al., 1997; Camarero et al., 2012; Abdelaziz et al., 2016). Although bacterial strains are also being explored for lignin biotransformation, the β-ketoadipate pathway is one of the primary pathways for aromatic derivative cleavage and is described mostly in prokaryotes, and therefore, there is much to be discovered about metabolic pathways and the enzymes responsible for lignin bioconversion (Linger et al., 2014; T. Li and Takkellapati, 2018). A better understanding of lignin derivative metabolism is crucial for industrial implementation and the production of high value-added chemicals.

In this context, the appropriate choice for screening methodologies to exploit microbial biodiversity and perform enzyme selection is essential. Also, it is important to emphasize that the utilization of multiple screening methodologies may give a complete arsenal of lignolytic activities in a given sample. Sequence-based methodologies represent a powerful approach to identifying the genetic repertoire and possible metabolic strategies associated with ligninolytic microbes. Although the ligninolytic activity has been established for fungi, the genetic bases associated with lignin breakdown is still not well understood, and it is still more often reported in bacteria (Ravi et al., 2017). Studies focused on the association between genetic variance, and ligninolytic potential are still scarce, justifying the need to sequence and establish ligninolytic microbial models and deepen our studies of the already-sequenced genomes available in databases. Thus, in combination with other omics techniques, DNA sequencing represents a promising strategy for discovering important genes related to lignin depolymerization. Culture-based methods such as ABTS and chromogenic substrates are the best-established ones for identifying lignin-degrading strains as well as ligninolytic enzymes (Achar et al., 2014; Kumar et al., 2017). In addition to the difficulties in isolating some strains and maintaining their viability under laboratory conditions, these approaches are still beneficial when coupled with analytical tools, giving a complete overview of lignin biotransformation and may continue being used as an initial screening method when a given microbial culture collection is available. Finally, the use of biosensors such as SIGEX and GESS is rising to overcome cultivation obstacles and to allow isolation of sequence-independent activities, enabling the harnessing of metagenomic samples in poorly explored environments and microbiomes. In addition, lignin derivative production by industrial strains is being monitored by these biosensors (Helm et al., 2018). Overall, the methods shown here are pivotal for gaining a better understanding of lignin degradation and broadening horizons for the identification of new biocatalysts, enzymes, and even genes that could be used in biotechnological strategies for lignin conversion.

## Author Contributions

CG wrote the introduction and biosensor sections. TB wrote the sequence-based methodologies. CS and EF collected all the globally available data on lignin and wrote the “Introduction and Conclusion” section regarding lignin utilization. EN wrote activity-based methodologies for the isolation of ligninolytic activities. MC and NP planned the entire review and reviewed the manuscript.

## Conflict of Interest

The authors declare that the research was conducted in the absence of any commercial or financial relationships that could be construed as a potential conflict of interest.

## Footnotes

1 http://www.fao.org/faostat/en/#data

2 www.cazy.org/

## Supplementary Material

DATA SHEET S1 Raw data for global distribution of lignin.

## Abbreviations

AA: auxiliary activities
DDVA: 5,5 Dehydrodivanillic acid
EGFP: enhanced green fluorescent protein
FACS: fluorescence-activated cell sorting
GC/MS: gas chromatography coupled to mass spectrometry
GESS: genetic enzyme screening system
GGCE: guaiacylglycerol- β-O -4-guaiacyl ether
LC/MS: liquid chromatography coupled to mass spectrometry
LME: lignin-modifying enzymes
LMW: low molecular weight
LPMO: lytic polysaccharide monooxygenases
MnP: manganese peroxidases
MPH: methyl parathion hydrolases
MWL: milled wood lignin
NAD: nicotinamide adenine dinucleotide
NIMS: nanostructure-initiator mass spectrometry
PGPA: prokaryotic genome annotation pipeline
RAST: rapid subsystem technology
SIGEX: substrate-induced gene expression
SPORL: sulfite pretreatment to overcome recalcitrance of lignocellulose
TCA: tricarboxylic acid cycle
TL: technical lignin
YFP: yellow fluorescent protein.

## References

[B1] Abdelaziz O. Y. Daniel P. Brink J. P. Krithika R. Mingzhe S. Javier G.-H. (2016). Biological valorization of low molecular weight lignin. *Biotechnol. Adv.* 34 1318–1346. 10.1016/j.biotechadv.2016.10.001 27720980

[B2] Achar R. R. Venkatesh B. K. Sharanappa P. Priya B. S. Nanjunda Swamy S. (2014). Evidence for peroxidase activity in caralluma umbellata. *Appl. Biochem. Biotechnol.* 173 1955–1962. 10.1007/s12010-014-1013-0 24943097

[B3] Adhi T. P. Korus R. A. Crawford D. L. (1989). Production of major extracellular enzymes during lignocellulose degradation by two streptomycetes in agitated submerged culture. *Appl. Environ. Microbiol.* 55 1165–1168. 16347909 10.1128/aem.55.5.1165-1168.1989 PMC184271

[B4] Ahmad M. Joseph N. R. Elizabeth M. H. Rahul S. Lindsay D. E. Timothym R. (2011). Identification of DypB from *Rhodococcus jostii* RHA1 as a lignin peroxidase. *Biochemistry* 50 5096–5107. 10.1021/bi101892z 21534568

[B5] Alcalde M. Thomas B. Zumárraga M. García-Arellano H. Mencía M. Francisco J. P. (2005). Screening mutant libraries of fungal laccases in the presence of organic solvents. *J. Biomol. Screen.* 10 624–631. 10.1177/1087057105277058 16103414

[B6] Altschul S. F. Gish W. Miller W. Myers E. W. Lipman D. J. (1990). Basic local alignment search tool. *J. Mol. Biol.* 215 403–410. 2231712 10.1016/S0022-2836(05)80360-2

[B7] Artimo P. Jonnalagedda M. Arnold K. Baratin D. Csardi G. de Castro E. (2012). ExPASy: SIB bioinformatics resource portal. *Nucleic Acids Res.* 40 W597–W603. 10.1093/nar/gks400 22661580 PMC3394269

[B8] Aziz R. K. Bartels D. Aaron A. Best R. DeJongh M. Robert A. E. (2008). The RAST server: rapid annotations using subsystems technology. *BMC Genomics* 9:75. 10.1186/1471-2164-9-75 18261238 PMC2265698

[B9] Bajwa D. S. Pourhashem G. Ullah A. H. Bajwa S. G. (2019). A concise review of current lignin production, applications, products and their environmental impact. *Ind. Crops Prod.* 139:111526 10.1016/j.indcrop.2019.111526

[B10] Bandounas L. Nick J. Wierckx J. H. de Winde H. Ruijssenaars H. (2011). Isolation and characterization of novel bacterial strains exhibiting ligninolytic potential. *BMC Biotechnol.* 11:94. 10.1186/1472-6750-11-94 21995752 PMC3212925

[B11] Becker H. Gärtner C. (2012). Microfluidics and the life sciences. *Sci. Prog.* 95(Pt 2), 175–198. 10.3184/003685012x13361524970266 22893979 PMC10365460

[B12] Becker J. Wittman C. (2019). A field of dreams: lignin valorization into chemicals, materials, fuels, and health-care products. *Biotechnol. Adv.* 37:107360. 10.1016/j.biotechadv.2019.02.016 30959173

[B13] Beckham G. T. Christopher W. Johnson E. M. Karp M. Salvachúa D. Derek R. V. (2016). Opportunities and challenges in biological lignin valorization. *Curr. Opin. Biotechnol.* 42 40–53. 10.1016/j.copbio.2016.02.030 26974563

[B14] Behling R. Valange S. Chatel G. (2016). Heterogeneous catalytic oxidation for lignin valorization into valuable chemicals: what results? What limitations? What trends? *Green Chem.* 18 1839–1854. 10.1039/c5gc03061g

[B15] Berman H. M. Westbrook J. Feng Z. Gilliland G. Bhat T. N. Weissig H. I. (2000). The protein data bank. *Nucleic Acids Res.* 28 235–242. 10592235 10.1093/nar/28.1.235 PMC102472

[B16] Bey M. Zhou S. Poidevin L. Henrissat B. Pedro M. C. Sigoillot J.-C. (2013). Cello-oligosaccharide oxidation reveals differences between two lytic polysaccharide monooxygenases (family GH61) from Podospora anserina. *Appl. Environ. Microbiol.* 79 488–496. 10.1128/AEM.02942-12 23124232 PMC3553762

[B17] Bomble Y. J. Lin C. Amore A. Wei H. Evert K. H. Ciesielski P. N. (2017). Lignocellulose deconstruction in the biosphere. *Curr. Opin. Chem. Biol.* 41 61–70. 29100023 10.1016/j.cbpa.2017.10.013

[B18] Bousse L. (1995). Whole cell biosensors. *Sens. Actuators B Chem.* 34 483–486. 10.1109/sensor.1995.721853

[B19] Brink D. P. Krithika R. Gunnar L. Marie F. (2019). Mapping the diversity of microbial lignin catabolism: experiences from the eLignin database. *Appl. Microbiol. Biotechnol.* 103 3979–4002. 10.1007/s00253-019-09692-4 30963208 PMC6486533

[B20] Brown M. E. Tiago B. Michelle C. Chang Y. (2012). Identification and characterization of a multifunctional dye peroxidase from a lignin-reactive bacterium. *ACS Chem. Biol.* 7 2074–2081. 10.1021/cb300383y 23054399

[B21] Brown M. E. Walker M. C. Nakashige T. G. Iavarone A. T. Chang Y. (2011). Discovery and characterization of heme enzymes from unsequenced bacteria: application to microbial lignin degradation. *J. Am. Chem. Soc.* 133 18006–18009. 10.1021/ja203972q 21671563

[B22] Bruce T. Martinez I. B. Neto O. Vicente A. Kruger R. Thompson F. L. (2010). Bacterial community diversity in the Brazilian Atlantic forest soils. *Microb. Ecol.* 60 840–849. 10.1007/s00248-010-9750-2 20886336

[B23] Bugg T. D. H. Ahmad M. Hardiman E. M. Rahmanpour R. (2012). Pathways for degradation of lignin in bacteria and fungi. *Nat. Prod. Rep.* 28 1883–1896. 10.1039/c1np00042j 21918777

[B24] Bugg T. D. H. Rahmanpour R. (2015). Enzymatic conversion of lignin into renewable chemicals. *Curr. Opin. Chem. Biol.* 29 10–17. 10.1016/j.cbpa.2015.06.009 26121945

[B25] Camarero S. Pardo I. Cañas A. I. Molina P. Record E. Martínez A. T. (2012). Engineering platforms for directed evolution of laccase from pycnoporus cinnabarinus. *Appl. Environ. Microbiol.* 78 1370–1384. 10.1128/AEM.07530-11 22210206 PMC3294479

[B26] Cantarel B. L. Coutinho P. M. Rancurel C. Bernard T. Lombard V. Henrissat B. (2009). The carbohydrate-active enzymes database (CAZy): an expert resource for glycogenomics. *Nucleic Acids Res.* 37 D233–D238. 10.1093/nar/gkn663 18838391 PMC2686590

[B27] Choi S.-L. Rha E. Lee S. Kim H. Kwon K. Jeong Y. (2014). Toward a generalized and high-throughput enzyme screening system based on artificial genetic circuits. *ACS Synth. Biol.* 3 163–171. 10.1021/sb400112u 24295047

[B28] Chong G.-G. Huang X.-J. Di J.-H. Xu D.-Z. He Y.-C. Pei Y.-N. (2018). Biodegradation of alkali lignin by a newly isolated *Rhodococcus pyridinivorans* CCZU-B16. *Bioprocess Biosyst. Eng.* 41 501–510. 10.1007/s00449-017-1884-x 29279999

[B29] Cragg S. M. Beckham G. T. Bruce N. C. Bugg T. D. H. Distel D. Dupree P. (2015). Lignocellulose degradation mechanisms across the tree of life. *Curr. Opin. Chem. Biol.* 29 108–119. 10.1016/j.cbpa.2015.10.018 26583519 PMC7571853

[B30] Curtis T. P. Sloan W. T. (2004). Prokaryotic diversity and its limits: microbial community structure in nature and implications for microbial ecology. *Curr. Opin. Microbiol.* 7 221–226. 10.1016/j.mib.2004.04.010 15196488

[B31] Daioglou V. Stehfest E. Wicke B. Faaij A. van Vuuren D. P. (2016). Projections of the availability and cost of residues from agriculture and forestry. *GCB Bioenergy* 8 456–470. 10.1111/gcbb.12285

[B32] Davis J. R. Goodwin L. A. Woyke T. Teshima H. Bruce D. Detter C. (2012). Genome sequence of *Amycolatopsis* Sp. strain ATCC 39116, a plant biomass-degrading actinomycete. *J. Bacteriol.* 194 2396–2397. 10.1128/JB.00186-12 22493203 PMC3347045

[B33] Davis J. R. Lynne G. Hazuki T. Detter C. Tapia R. Huntemann M. (2013). Genome sequence of *Streptomyces viridosporus* strain T7A ATCC 39115, a lignin-degrading actinomycete. *Genome Announc.* 1:e00416-13. 10.1128/genomeA.00416-13 23833133 PMC3703594

[B34] Deng K. Zeng J. Cheng G. Gao J. Sale K. L. Blake A. (2018). Rapid characterization of the activities of lignin-modifying enzymes based on nanostructure-initiator mass spectrometry (NIMS). *Biotechnol. Biofuels* 11:266. 10.1186/s13068-018-1261-2 30275906 PMC6158898

[B35] Eddy S. R. (2009). A new generation of homology search tools based on probabilistic inference. *Genome Inform.* 23 205–211. 20180275

[B36] Eggeling L. Bott M. Marienhagen J. (2015). Novel screening methods—biosensors. *Curr. Opin. Biotechnol.* 35 30–36. 10.1016/j.copbio.2014.12.021 25578902

[B37] Fiorentino G. Ronca R. Bartolucci S. (2009). A novel *E. coli* biosensor for detecting aromatic aldehydes based on a responsive inducible archaeal promoter fused to the green fluorescent protein. *Appl. Microbiol. Biotechnol.* 82 67–77. 10.1007/s00253-008-1771-0 18998120

[B38] Food and Agriculture Organization of the United Nations (2019). *Database (FAOSTAT).* Available online at: http://www.fao.org/faostat/en/#data (accessed January 5, 2019).

[B39] Fritzsch F. S. O. Dusny C. Frick O. Schmid A. (2012). Single-cell analysis in biotechnology, systems biology, and biocatalysis. *Annu. Rev. Chem. Biomol. Eng.* 3 129–155. 10.1146/annurev-chembioeng-062011-081056 22468600

[B40] Gerlt J. A. Babbitt P. C. (2001). Divergent evolution of enzymatic function: mechanistically diverse superfamilies and functionally distinct suprafamilies. *Annu. Rev. Biochem.* 70 209–246. 10.1146/annurev.biochem.70.1.209 11395407

[B41] Gillet S. Aguedo M. Petitjean L. Morais A. R. C. da Costa Lopes A. M. Łukasik R. M. (2017). Lignin transformations for high value applications: towards targeted modifications using green chemistry. *Green Chem.* 19 4200–4233. 10.1039/c7gc01479a

[B42] Granja-Travez R. S. Bugg T. D. H. (2018). Characterization of multicopper oxidase CopA from *Pseudomonas* Putida KT2440 and *Pseudomonas* Fluorescens Pf-5: involvement in bacterial lignin oxidation. *Arch. Biochem. Biophys.* 660 97–107. 10.1016/j.abb.2018.10.012 30347180

[B43] Grumaz C. Rais D. Kirstahler P. Vainshtein Y. Rupp S. Zibek S. (2017). Draft genome sequence of *Pseudonocardia autotrophica* strain DSM 43083, an efficient producer of peroxidases for lignin modification. *Genome Announc.* 5:e01562-16. 10.1128/genomeA.01562-16 28153904 PMC5289690

[B44] Gui Q. Lawson T. Shan S. Yan L. Liu Y. (2017). The application of whole cell-based biosensors for use in environmental analysis and in medical diagnostics. *Sensors* 17:1623. 10.3390/s17071623 28703749 PMC5539819

[B45] Guillén F. Martínez M. J. Muñoz C. Martínez A. T. (1997). Quinone redox cycling in the ligninolytic fungus *Pleurotus eryngii* leading to extracellular production of superoxide anion radical. *Arch. Biochem. Biophys.* 339 190–199. 10.1006/abbi.1996.9834 9056249

[B46] Gutiérrez A. Caramelo L. Prieto A. Martínez M. J. Martínez A. T. (1994). Anisaldehyde production and aryl-alcohol oxidase and dehydrogenase activities in ligninolytic fungi of the genus pleurotus. *Appl. Environ. Microbiol.* 60 1783–1788. 8031078 10.1128/aem.60.6.1783-1788.1994 PMC201562

[B47] Hämäläinen V. Grönroos T. Suonpää A. Heikkilä M. W. Romein B. Ihalainen P. (2018). Enzymatic processes to unlock the lignin value. *Front. Bioeng. Biotechnol.* 6:20. 10.3389/fbioe.2018.00020 29623274 PMC5874288

[B48] Helm E. Genee J. Sommer M. O. A. (2018). The evolving interface between synthetic biology and functional metagenomics. *Nat. Chem. Biol.* 14 752–759. 10.1038/s41589-018-0100-x 30013060

[B49] Hinz U. The UniProt Consortium (2010). From protein sequences to 3D-structures and beyond: the example of the Uniprot knowledgebase. *Cell. Mol. Life Sci.* 67 1049–1064. 10.1007/s00018-009-0229-6 20043185 PMC2835715

[B50] Ho J. C. H. Sandip V. P. Steven J. H. Yadav V. G. (2018). An improved whole-cell biosensor for the discovery of lignin-transforming enzymes in functional metagenomic screens. *ACS Synth. Biol.* 7 392–398. 10.1021/acssynbio.7b00412 29182267

[B51] Horn S. J. Vaaje-Kolstad G. Westereng B. Eijsink V. (2012). Novel enzymes for the degradation of cellulose. *Biotechnol. Biofuels* 5:45. 10.1186/1754-6834-5-45 22747961 PMC3492096

[B52] Hu J. Zhang Q. Lee D.-J. (2018). Kraft lignin biorefinery: a perspective. *Bioresour. Technol.* 247 1181–1183. 10.1016/j.biortech.2017.08.169 28899675

[B53] Huang L. Zhang H. Wu P. Entwistle S. Li X. Yohe T. (2018). dbCAN-Seq: a database of carbohydrate-active enzyme (CAZyme) sequence and annotation. *Nucleic Acids Res.* 46 D516–D521. 10.1093/nar/gkx894 30053267 PMC5753378

[B54] Huang X.-F. Santhanam N. Badri D. V. Hunter W. Manter D. K. Decker S. R. (2013). Isolation and characterization of lignin-degrading bacteria from rainforest soils. *Biotechnol. Bioeng.* 110 1616–1626. 10.1002/bit.24833 23297115

[B55] Hughes J. B. Hellmann J. J. Ricketts T. H. Bohannan B. J. (2001). Counting the uncountable: statistical approaches to estimating microbial diversity. *Appl. Environ. Microbiol.* 67 4399–4406. 10.1128/aem.67.10.4399-4406.2001 11571135 PMC93182

[B56] Jeong Y.-S. Choi S.-L. Kyeong H.-H. Kim J.-H. Kim E.-J. Pan J.-G. (2012). High-throughput screening system based on phenolics-responsive transcription activator for directed evolution of organophosphate-degrading enzymes. *Protein Eng. Des. Sel.* 25 725–731. 10.1093/protein/gzs071 23077277

[B57] Kadam K. L. McMillan J. D. (2003). Availability of corn stover as a sustainable feedstock for bioethanol production. *Bioresour. Technol.* 88 17–25. 10.1016/s0960-8524(02)00269-9 12573559

[B58] Kakkar S. Bais S. (2014). A review on protocatechuic acid and its pharmacological potential. *ISRN Pharmacol.* 2014:952943. 10.1155/2014/952943 25006494 PMC4005030

[B59] Kameshwar A. K. S. Barber R. Qin W. (2018). Comparative modeling and molecular docking analysis of white, brown and soft rot fungal laccases using lignin model compounds for understanding the structural and functional properties of laccases. *J. Mol. Graph. Model.* 79 15–26. 10.1016/j.jmgm.2017.10.019 29127854

[B60] Kameshwar A. K. S. Qin W. (2018). Comparative study of genome-wide plant biomass-degrading CAZymes in white rot, brown rot and soft rot fungi. *Mycology* 9 93–105. 10.1080/21501203.2017.1419296 30123665 PMC6059041

[B61] Kanehisa M. Goto S. Sato Y. Kawashima M. Furumichi M. Tanabe M. (2014). Data, information, knowledge and principle: back to metabolism in KEGG. *Nucleic Acids Res.* 42 D199–D205. 10.1093/nar/gkt1076 24214961 PMC3965122

[B62] Kanehisa M. Sato Y. Kawashima M. Furumichi M. Tanabe M. (2016). KEGG as a reference resource for gene and protein annotation. *Nucleic Acids Res.* 44 D457–D462. 10.1093/nar/gkv1070 26476454 PMC4702792

[B63] Kohlstedt M. Starck S. Barton N. Stolzenberger J. Selzer M. Mehlmann K. (2018). From lignin to nylon: cascaded chemical and biochemical conversion using metabolically engineered *Pseudomonas* Putida. *Metab. Eng.* 47 279–293. 10.1016/j.ymben.2018.03.003 29548984

[B64] Koua D. Cerutti L. Falquet L. Sigrist C. J. A. Theiler G. Hulo N. (2009). PeroxiBase: a database with new tools for peroxidase family classification. *Nucleic Acids Res.* 37 D261–D266. 10.1093/nar/gkn680 18948296 PMC2686439

[B65] Kudo S. Harada A. Kubota H. Sasaki K. Kaneta T. (2017). Simultaneous determination of manganese peroxidase and lignin peroxidase by capillary electrophoresis enzyme assays. *ACS Omega* 10 7329–7333. 10.1021/acsomega.7b00998 31457306 PMC6645401

[B66] Kumar A. Singh D. Krishna K. S. Sakshi A. Amarjeet K. S. Gill S. S. (2017). Gel-based purification and biochemical study of laccase isozymes from *Ganoderma* Sp. and its role in enhanced cotton callogenesis. *Front. Microbiol.* 8:674. 10.3389/fmicb.2017.00674 28473815 PMC5397484

[B67] Lee J. (1997). Biological conversion of lignocellulosic biomass to ethanol. *J. Biotechnol.* 56 1–24. 10.1016/s0168-1656(97)00073-4 9246788

[B68] Lee K. H. Wi S. Singh A. P. Kim Y. S. (2004). Micromorphological characteristics of decayed wood and laccase produced by the brown-rot fungus *Coniophora puteana*. *J. Wood Sci.* 50 281–284.

[B69] Levasseur A. Elodie D. Vincent L. Pedro M. C. Bernard H. (2013). Expansion of the enzymatic repertoire of the CAZy database to integrate auxiliary redox enzymes. *Biotechnol. Biofuels* 6:41. 10.1186/1754-6834-6-41 23514094 PMC3620520

[B70] Li T. Takkellapati S. (2018). The current and emerging sources of technical lignins and their applications. *Biofuel. Bioprod. Biorefin.* 12 756–787. 10.1002/bbb.1913 30220952 PMC6134873

[B71] Li X. William T. B. Phillips C. M. Marletta M. A. Jamie H. D. (2012). Structural basis for substrate targeting and catalysis by fungal polysaccharide monooxygenases. *Structure* 20 1051–1061. 10.1016/j.str.2012.04.002 22578542 PMC3753108

[B72] Lima B. Maria J. Jiménez D. J. Cortes-Tolalpa L. Elsas J. D. (2016). Soil-derived microbial consortia enriched with different plant biomass reveal distinct players acting in lignocellulose degradation. *Microb. Ecol.* 71 616–627. 10.1007/s00248-015-0683-7 26487437 PMC4788684

[B73] Linger J. G. Vardon D. R. Guarnieri M. T. Karp E. M. Hunsinger G. B. Franden M. (2014). Lignin valorization through integrated biological funneling and chemical catalysis. *Proc. Natl. Acad. Sci. U.S.A.* 111 12013–12018. 10.1073/pnas.1410657111 25092344 PMC4143016

[B74] Liu Z.-H. Xie S. Lin F. Jin M. Yuan J. S. (2018). Combinatorial pretreatment and fermentation optimization enabled a record yield on lignin bioconversion. *Biotechnol. Biofuels* 11:21. 10.1186/s13068-018-1021-3 29422949 PMC5787925

[B75] Lombard V. Ramulu H. G. Drula E. Coutinho P. M. Bernard H. (2014). The carbohydrate-active enzymes database (CAZy) in 2013. *Nucleic Acids Res.* 42 D490–D495. 10.1093/nar/gkt1178 24270786 PMC3965031

[B76] Mannan A. A. Liu D. Zhang F. Oyarzún D. A. (2017). Fundamental design principles for transcription-factor-based metabolite biosensors. *ACS Synth. Biol.* 6 1851–1859. 10.1021/acssynbio.7b00172 28763198

[B77] Martínez A. T. Mariela S. Ruiz-Dueñas F. J. Ferreira P. Camarero S. Guillén F. (2005). Biodegradation of lignocellulosics: microbial, chemical, and enzymatic aspects of the fungal attack of lignin. *Int. Microbiol.* 8 195–204. 16200498

[B78] Martínez A. T. (2002). Molecular biology and structure-function of lignin-degrading heme peroxidases. *Enzyme Microb. Technol.* 30 425–444. 10.1016/s0141-0229(01)00521-x

[B79] Masai E. Katayama Y. Nishikawa S. Fukuda M. (1999). Characterization of *Sphingomonas paucimobilis* SYK-6 genes involved in degradation of lignin-related compounds. *J. Ind. Microbiol. Biotechnol.* 23 364–373. 10.1038/sj.jim.2900747 11423957

[B80] McLeod M. P. Warren R. L. Hsiao W. W. L. Araki N. Myhre M. Fernandes C. (2006). The complete genome of *Rhodococcus* Sp. RHA1 provides insights into a catabolic powerhouse. *Proc. Natl. Acad. Sci. U.S.A.* 103 15582–15587. 10.1073/pnas.0607048103 17030794 PMC1622865

[B81] Melo-Nascimento A. O. Dos S. Claudia T. Neves C. Andrade E. Andrade A. C. (2018). Functional characterization of ligninolytic *Klebsiella* Spp. strains associated with soil and freshwater. *Arch. Microbiol.* 200 1267–1278. 10.1007/s00203-018-1532-0 29947838

[B82] Mercer D. K. Iqbal M. Miller P. McCarthy A. J. (1996). Screening actinomycetes for extracellular peroxidase activity. *Appl. Environ. Microbiol.* 62 2186–2190. 16535344 10.1128/aem.62.6.2186-2190.1996 PMC1388882

[B83] Monssef R. Abd El A. Rehan A. Monssef A. Hassan E. Ramadan E. M. (2016). Production of laccase enzyme for their potential application to decolorize fungal pigments on aging paper and parchment. *Ann. Agric. Sci.* 61 145–154. 10.1016/j.aoas.2015.11.007

[B84] Moraes E. C. Alvarez T. M. Persinoti G. F. Tomazetto G. Brenelli L. B. Douglas A. (2018). Lignolytic-consortium omics analyses reveal novel genomes and pathways involved in lignin modification and valorization. *Biotechnol. Biofuels* 11:75. 10.1186/s13068-018-1073-4 29588660 PMC5863372

[B85] Motato-Vásquez V. Pires R. M. Valle Vitali V. M. de Mello Gugliotta A. (2016). Cultural and ligninolytic activity studies of some polypores (Basidiomycota) from Brazilian Atlantic forest, São Paulo State, Brazil. *Hoehnea* 43 289–300. 10.1590/2236-8906-81/2015

[B86] Naseem A. Tabasum S. Zia K. M. Zuber M. Ali M. Noreen A. (2016). Lignin-derivatives based polymers, blends and composites: a review. *Int. J. Biol. Macromol.* 93(Pt A), 296–313. 10.1016/j.ijbiomac.2016.08.030 27521847

[B87] Nikel P. I. de Lorenzo V. (2018). *Pseudomonas putida* as a functional chassis for industrial biocatalysis: from native biochemistry to trans-metabolism. *Metab. Eng.* 50 142–155. 10.1016/j.ymben.2018.05.005 29758287

[B88] Nishikawa S. Sonoki T. Kasahara T. Obi T. Kubota S. Kawai S. (1998). Cloning and sequencing of the *Sphingomonas* (*Pseudomonas*) *paucimobilis* gene essential for the O demethylation of vanillate and syringate. *Appl. Environ. Microbiol.* 64 836–842. 9501423 10.1128/aem.64.3.836-842.1998 PMC106335

[B89] Northen T. R. Lee J.-C. Hoang L. Raymond J. Hwang D.-R. Yannone S. M. (2008). A nanostructure-initiator mass spectrometry-based enzyme activity assay. *Proc. Natl. Acad. Sci. U.S.A.* 105 3678–3683. 10.1073/pnas.0712332105 18319341 PMC2268803

[B90] Nozaki K. Beh C. H. Mizuno M. Isobe T. Shiroishi M. Kanda T. (2008). Screening and investigation of dye decolorization activities of basidiomycetes. *J. Biosci. Bioeng.* 105 69–72. 10.1263/jbb.105.69 18295724

[B91] Ohta Y. Nishi S. Haga T. Tsubouchi T. Hasegawa R. Konishi M. (2012). Screening and phylogenetic analysis of deep-sea bacteria capable of metabolizing lignin-derived aromatic compounds. *Open J. Mar. Sci.* 2 177–187. 10.4236/ojms.2012.24021

[B92] Pereira J. H. Richard A. H. Gall D. L. McAndrew R. P. Deng K. Holland K. C. (2016). Structural and biochemical characterization of the early and late enzymes in the lignin β-aryl ether cleavage pathway from *Sphingobium* Sp. SYK-6. *J. Biol. Chem.* 291 10228–10238. 10.1074/jbc.M115.700427 26940872 PMC4858972

[B93] Pevsner J. (2009). *Bioinformatics and Functional Genomics*, 2nd Edn Hoboken, NJ: John Wiley and Sons 10.1002/9780470451496

[B94] Picart P. de María P. D. Schallmey A. (2015). From gene to biorefinery: microbial β-etherases as promising biocatalysts for lignin valorization. *Front. Microbiol.* 6:916. 10.3389/fmicb.2015.00916 26388858 PMC4560021

[B95] Ponnusamy V. K. Nguyen D. D. Dharmaraja J. Shobana S. Banu J. Saratale R. G. (2019). A review on lignin structure, pretreatments, fermentation reactions and biorefinery potential. *Bioresour. Technol.* 271 462–472. 10.1016/j.biortech.2018.09.070 30270050

[B96] Prabhakaran M. Couger M. B. Jackson C. A. Weirick T. Fathepure B. Z. (2015). Genome sequences of the lignin-degrading *Pseudomonas* Sp. strain YS-1p and *Rhizobium* Sp. strain YS-1r isolated from decaying wood. *Genome Announc.* 3:e00019-15. 10.1128/genomeA.00019-15 25744986 PMC4358373

[B97] Quinlan R. J. Matt D. S. Leila L. L. Otten H. Poulsen J.-C. Johansen K. S. (2011). Insights into the oxidative degradation of cellulose by a copper metalloenzyme that exploits biomass components. *Proc. Natl. Acad. Sci. U.S.A.* 108 15079–15084. 10.1073/pnas.1105776108 21876164 PMC3174640

[B98] Ragauskas A. J. Beckham G. T. Biddy M. J. Chandra R. Chen F. Davis M. F. (2014). Lignin valorization: improving lignin processing in the biorefinery. *Science* 344:1246843. 10.1126/science.1246843 24833396

[B99] Raj A. Krishna Reddy M. M. Chandra R. Purohit H. J. Kapley A. (2007). Biodegradation of Kraft-lignin by *Bacillus* Sp. isolated from sludge of pulp and paper mill. *Biodegradation* 18 783–792. 10.1007/s10532-007-9107-9 17308883

[B100] Ravi K. García-Hidalgo J. Gorwa-Grauslund M. F. Lidén G. (2017). Conversion of lignin model compounds by *Pseudomonas putida* KT2440 and isolates from compost. *Appl. Microbiol. Biotechnol.* 101 5059–5070. 10.1007/s00253-017-8211-y 28299400 PMC5486835

[B101] Rinaldi R. Jastrzebski R. Clough M. T. Ralph J. Kennema M. Pieter C. A. (2016). Paving the way for lignin valorisation: recent advances in bioengineering, biorefining and catalysis. *Angew. Chem. Int. Ed.* 55 8164–8215. 10.1002/anie.201510351 27311348 PMC6680216

[B102] Rothschild N. Levkowitz A. Hadar Y. Dosoretz C. (1999). Extracellular mannose-6-phosphatase of *Phanerochaete chrysosporium*: a lignin peroxidase-modifying enzyme. *Arch. Biochem. Biophys.* 372 107–111. 10.1006/abbi.1999.1474 10562422

[B103] Sana B. Chia K. H. B. Raghavan S. S. Ramalingam B. Nagarajan N. Seayad J. (2017). Development of a genetically programed vanillin-sensing bacterium for high-throughput screening of lignin-degrading enzyme libraries. *Biotechnol. Biofuels* 10:32. 10.1186/s13068-017-0720-5 28174601 PMC5291986

[B104] Santos E. de los L. C. Meyerowitz J. T. Mayo S. L. Murray R. M. (2016). Engineering transcriptional regulator effector specificity using computational design and in vitro rapid prototyping: developing a vanillin sensor. *ACS Synth. Biol.* 5 287–295. 10.1021/acssynbio.5b00090 26262913

[B105] Sayers E. W. Beck J. Brister J. R. Bolton E. E. Canese K. Comeau D. C. (2020). Database resources of the national center for biotechnology information. *Nucleic Acids Res.* 48 D9–D16. 10.1093/nar/gkz899 31602479 PMC6943063

[B106] Schmid A. Neubauer P. (2010). Analytical biotechnology: from single molecule and single cell analyses to population dynamics of metabolites and cells. *Curr. Opin. Biotechnol.* 21 1–3. 10.1016/j.copbio.2010.02.010 20189376

[B107] Seemann T. (2014). Prokka: rapid prokaryotic genome annotation. *Bioinformatics* 30 2068–2069. 10.1093/bioinformatics/btu153 24642063

[B108] Siedler S. Khatri N. K. Zsohár A. Kjærbølling I. Vogt M. Hammar P. (2017). Development of a bacterial biosensor for rapid screening of yeast P-coumaric acid production. *ACS Synth. Biol.* 6 1860–1869. 10.1021/acssynbio.7b00009 28532147

[B109] Silva C. O. G. Raissa P. V. Filho E. X. F. (2018). Bringing plant cell wall-degrading enzymes into the lignocellulosic biorefinery concept. *Biofuel. Bioprod. Biorefin.* 12 277–289. 10.1002/bbb.1832

[B110] Strassberger Z. Tanase S. Rothenberg G. (2014). The pros and cons of lignin valorisation in an integrated biorefinery. *RSC Adv.* 4 25310–25318. 10.1039/c4ra04747h

[B111] Suman S. K. Dhawaria M. Tripathi D. Kumar P. Pankaj K. K. (2016). Investigation of lignin biodegradation by *Trabulsiella* Sp. isolated from termite gut. *Int. Biodeterior. Biodegradation* 112 12–17. 10.1016/j.ibiod.2016.04.036

[B112] Sun X. Zhang R. Zhang Y. (2004). Production of lignocellulolytic enzymes by *Trametes gallica* and detection of polysaccharide hydrolase and laccase activities in polyacrylamide gels. *J. Basic Microbiol.* 44 220–231. 10.1002/jobm.200310376 15162396

[B113] Sun Z. Fridrich B. de Santi A. Elangovan S. Barta K. (2018). Bright side of lignin depolymerization: toward new platform chemicals. *Chem. Rev.* 118 614–678. 10.1021/acs.chemrev.7b00588 29337543 PMC5785760

[B114] Tatusova T. DiCuccio M. Badretdin A. Chetvernin V. Nawrocki E. P. Zaslavsky L. (2016). NCBI prokaryotic genome annotation pipeline. *Nucleic Acids Res.* 44 6614–6624. 10.1093/nar/gkw569 27342282 PMC5001611

[B115] Taylor C. R. Hardiman E. M. Ahmad M. Sainsbury P. D. Norris P. R. Bugg T. D. H. (2012). Isolation of bacterial strains able to metabolize lignin from screening of environmental samples. *J. Appl. Microbiol.* 113 521–530. 10.1111/j.1365-2672.2012.05352.x 22642383

[B116] Temp U. Eggert C. Eriksson K. E. (1998). A small-scale method for screening of lignin-degrading microorganisms. *Appl. Environ. Microbiol.* 64 1548–1549. 16349553 10.1128/aem.64.4.1548-1549.1998 PMC106186

[B117] Tian J.-H. Pourcher A.-M. Bouchez T. Gelhaye E. Peu P. (2014). Occurrence of lignin degradation genotypes and phenotypes among prokaryotes. *Appl. Microbiol. Biotechnol.* 98 9527–9544. 10.1007/s00253-014-6142-4 25343973

[B118] Tomani P. (2010). The lignoboost process. *Cellulose Chem. Technol.* 44 53–58.

[B119] Uchiyama T. Abe T. Ikemura T. Watanabe K. (2005). Substrate-induced gene-expression screening of environmental metagenome libraries for isolation of catabolic genes. *Nat. Biotechnol.* 23 88–93. 10.1038/nbt1048 15608629

[B120] Uchiyama T. Miyazaki K. (2013). Metagenomic screening for aromatic compound-responsive transcriptional regulators. *PLoS One* 8:e75795. 10.1371/journal.pone.0075795 24098725 PMC3786939

[B121] Uchiyama T. Watanabe K. (2008). Substrate-induced gene expression (SIGEX) screening of metagenome libraries. *Nat. Protoc.* 3 1202–1212. 10.1038/nprot.2008.96 18600226

[B122] Vardon D. R. Rorrer N. A. Salvachúa D. Settle A. E. Johnson C. W. Menart M. J. (2016). cis,cis-muconic acid: separation and catalysis to bio-adipic acid for nylon-6,6 polymerization. *Green Chem.* 18 3397–3413. 10.1039/C5GC02844B

[B123] Vasilchenko L. G. Ludwig R. Yershevich O. P. Haltrich D. Rabinovich M. L. (2012). High-throughput screening for cellobiose dehydrogenases by prussian blue in situ formation. *Biotechnol. J.* 7 919–930. 10.1002/biot.201100480 22294389

[B124] Vitorino L. Bessa L. (2018). Microbial diversity: the gap between the estimated and the known. *Diversity* 10:46 10.3390/d10020046

[B125] Welker C. Balasubramanian V. Petti C. Rai K. DeBolt S. Mendu V. (2015). Engineering plant biomass lignin content and composition for biofuels and bioproducts. *Energies* 8 7654–7676. 10.3390/en8087654 26855670

[B126] Woo H. L. Nicholas R. B. Terry C. H. Julian L. F. Blake S. Karen D. (2014). Complete genome sequence of the lignin-degrading bacterium *Klebsiella* Sp. strain BRL6-2. *Stand. Genomic Sci.* 9:19. 10.1186/1944-3277-9-19 25566348 PMC4273726

[B127] Xu R. Zhang K. Liu P. Han H. Zhao S. Kakade A. (2018). Lignin depolymerization and utilization by bacteria. *Bioresour. Technol.* 269 557–566. 30219494 10.1016/j.biortech.2018.08.118

[B128] Xu Z. Lei P. Zhai R. Wen Z. Jin M. (2019). Recent advances in lignin valorization with bacterial cultures: microorganisms, metabolic pathways, and bio-products. *Biotechnol. Biofuels* 12:32. 10.1186/s13068-019-1376-0 30815030 PMC6376720

[B129] Xu Z. Qin L. Cai M. Hua W. Jin M. (2018). Biodegradation of Kraft lignin by newly isolated *Klebsiella pneumoniae*, *Pseudomonas putida*, and *Ochrobactrum tritici* strains. *Environ. Sci. Pollut. Res. Int.* 25 14171–14181. 10.1007/s11356-018-1633-y 29524172

[B130] Xue H. Shi H. Yu Z. He S. Liu S. Hou Y. (2014). Design, construction, and characterization of a set of biosensors for aromatic compounds. *ACS Synth. Biol.* 3 1011–1014. 10.1021/sb500023f 25524112

[B131] Yang C. Yue F. Cui Y. Xu Y. Shan Y. Liu B. (2018). Biodegradation of lignin by *Pseudomonas* Sp. Q18 and the characterization of a novel bacterial DyP-type peroxidase. *J. Indust. Microbiol. Biotechnol.* 45 913–927. 10.1007/s10295-018-2064-y 30051274

[B132] Zhang F. Keasling J. (2011). Biosensors and their applications in microbial metabolic engineering. *Trends Microbiol.* 19 323–329. 10.1016/j.tim.2011.05.003 21664818

[B133] Zhang Y.-H. (2008). Reviving the carbohydrate economy via multi-product lignocellulose biorefineries. *J. Ind. Microbiol. Biotechnol.* 35 367–375. 10.1007/s10295-007-0293-6 18180967

[B134] Zhang Z. Lai J. Wu K. Huang X. Guo S. Zhang L. (2018). Peroxidase-catalyzed chemiluminescence system and its application in immunoassay. *Talanta* 180 260–270. 10.1016/j.talanta.2017.12.024 29332809

[B135] Zhou H. Guo W. Xu B. Teng Z. Tao D. Lou Y. (2017). Screening and identification of lignin-degrading bacteria in termite gut and the construction of LiP-expressing recombinant *Lactococcus lactis*. *Microb. Pathog.* 112 63–69. 10.1016/j.micpath.2017.09.047 28943150

[B136] Zhu W. Westman G. Theliander H. (2014). Investigation and characterization of lignin precipitation in the lignoboost process. *J. Wood Chem. Technol.* 34 77–97. 10.1080/02773813.2013.838267

